# Exploring factors that mitigate the continued influence of misinformation

**DOI:** 10.1186/s41235-021-00335-9

**Published:** 2021-11-27

**Authors:** Irene P. Kan, Kendra L. Pizzonia, Anna B. Drummey, Eli J. V. Mikkelsen

**Affiliations:** Department of Psychological and Brain Sciences, Villanova University, 800 E. Lancaster Avenue, Villanova, PA 19085 USA

**Keywords:** Misinformation, continued influence effect, Situation model

## Abstract

**Background:**

The term “continued influence effect” (CIE) refers to the phenomenon that discredited and obsolete information continues to affect behavior and beliefs. The practical relevance of this work is particularly apparent as we confront fake news everyday. Thus, an important question becomes, how can we mitigate the continued influence of misinformation? Decades of research have identified several factors that contribute to the CIE reduction, but few have reported successful elimination. Across three studies, we evaluated the relative contribution of three factors (i.e., targeting the misinformation, providing an alternative explanation, and relative importance of the misinformation content) to the reduction of the CIE.

**Results:**

Across three studies and two different CIE measures, we found that alternative provision consistently resulted in CIE reduction. Furthermore, under certain conditions, the combination of alternative inclusion and direct targeting of misinformation in the correction statement resulted in successful elimination of the CIE, such that individuals who encountered that type of correction behaved similarly to baseline participants who never encountered the (mis)information. In contrast, under one CIE measure, participants who received correction statements that failed to include those elements referenced the (mis)information as frequently as baseline participants who never encountered a correction. Finally, we delineated several component processes involved in misinformation outdating and found that the extent of outdating success varied as a function of the causality of misinformation.

**Conclusions:**

The damaging effects of fake news are undeniable, and the negative consequences are exacerbated in the digital age. Our results contribute to our understanding of how fake news persists and how we may begin to mitigate their effects.

Misinformation can take many forms, ranging from an innocent misrepresentation to a blatant lie. Regardless of intent, the damage that misinformation can do is undeniable. Consequently, it is crucial to identify factors that perpetuate fake news and strategies that can mitigate their influence. One real-world example of such efforts comes from the Associated Press (AP), an American nonprofit news agency: To combat the spread of misinformation, AP features a weekly article called “Not Real News: A look at what didn’t happen this week” on their website. The preface of the online column reads, “A roundup of some of the most popular but completely untrue stories and visuals of the week. None of these are legit, even though they were shared widely on social media. The Associated Press checked them out. Here are the real facts” (https://apnews.com/NotRealNews). The reporter then proceeds to repeat the false claims and then counter them with the facts. The key question is: Are efforts such as these effective in minimizing the consequences of fake news? The importance of this question is amplified when we consider that approximately 93% of US adults get at least some of their news online, where information is spread rapidly (Pew Research Center, 2018).

Unfortunately, decades of research, both in the laboratory and in the field, present a rather grim picture for minimizing the damage caused by the spreading of misinformation (for reviews, see Lewandowsky et al., 2012; Rapp & Salovich, 2018). Here, we use the term “misinformation” to refer to content that is corrected or invalidated after its initial dissemination. Researchers have found that individuals’ beliefs, perceptions, and actions continue to be influenced by misinformation, suggesting that complete recovery from fake news is quite improbable once it has begun to spread (for reviews see Chan et al., 2017; Lewandowsky et al., 2012; Seifert, 2002; Walter & Tukachinsky, 2020). For example, before the invasion of Iraq in 2003, the Bush administration stressed the importance of removing Saddam Hussein from power by citing his probable stockpile of Weapons of Mass Destruction (WMDs). Although WMDs were never found, and the intelligence that supported their existence was later widely refuted, public opinion polls showed that approximately 20% of American adults still believed that Iraq had possessed a large collection of biological and chemical weapons (Lewandowsky et al., 2009). Researchers use the term “continued influence effect” (CIE) to describe the persistence of initially believed information (e.g., possession of WMDs), even when that information was later discredited (Johnson & Seifert, 1994).

Within the laboratory, the CIE is typically assessed with a text comprehension task, where information is presented incrementally, and readers are not allowed to backtrack to an earlier message. In one of the first CIE studies, Wilkes and Leatherbarrow (1988) presented individuals with a fictitious news story about a warehouse fire as a series of 13 time-stamped messages. The misinformation was presented toward the beginning of the story (message 5, stating that a closet contained paint cans and gas cylinders), and the correction was issued toward the end of the story (message 12, stating that the closet did not contain volatile materials and was in fact empty). After a short delay, comprehension questions were presented (e.g., “What was the possible cause of the toxic fumes?”), and responses that referred to the misinformation (e.g., burning of paint cans) were counted as evidence of the CIE. Approximately 30% of the participants in Wilkes and Leatherbarrow’s study continued to rely on the misinformation (Experiment 1). Importantly, almost all of these individuals (97%) correctly recalled the content of the correction, confirming that the CIE was not due to readers having forgotten the correction and thus lacking the most up-to-date information. In a separate demonstration of the effect, Johnson and Seifert (1994) found that over 90% of their subjects made at least one reference to volatile materials in the closet, suggesting that they failed to update their previous mental representation after they encountered the correction statement.

The CIE has been replicated and extended over the years, and a common theme that has emerged is that the CIE is extremely robust, in that it can be reliably induced and is extremely difficult to eliminate (Connor Desai & Reimers, 2019; Ecker et al., 2010, 2011a, b; Fein et al., 1997; Ithisuphalap et al., 2020; Lewandowsky et al., 2012; O’Rear & Radvansky, 2020; Wilkes & Leatherbarrow, 1988). Many studies have found that even when participants remember, understand, and believe the corrections aimed at retracting the misinformation, they remain susceptible to the CIE (see Lewandowsky et al., 2012 for a review). Here, we highlight several key findings from the various attempts to mitigate the CIE, and we will discuss them in the context of a situation (or mental) model of discourse.

As a narrative unfolds, an individual develops a situation model that represents the overall meaning and gist of the story and events, allowing the individual to keep track of what the narrative is about and supporting comprehension (Bailey & Zacks, 2015; Bower & Morrow, 1990; Johnson & Seifert, 1994; Johnson-Laird, 2012; Lewandowsky et al., 2012; van Oostendorp & Bonebakker, 1999; Wilkes & Leatherbarrow, 1988). The situation model is dynamic and evolving, in that new information becomes part of the model once it is encountered, and the ease of information integration depends on both narrative coherence and the extent to which the information aligns with an individual’s existing beliefs (Ecker et al., 2010; Ecker et al., 2014; Lewandowsky et al., 2009; Lewandowsky et al., 2005; but see Nyhan & Reifler, 2010). Furthermore, once a coherent narrative has been formed, it is largely resistant to updating, except when replacement information is available (Johnson-Laird, 2012; Verschueren et al., 2005). When alternative replacement information is presented, it allows a reader to disregard the initial discredited information and revise the mental model to include the alternative information (e.g., van Dijk & Kintsch, 1983). This two-step process is sometimes referred to as “outdating” (e.g., Kendeou et al., 2013; O’Brien et al., 2010).

Returning to misinformation, many researchers have characterized the CIE in the context of the situation model’s rapid development and resistance to revision, as evidenced by the ineffectiveness of immediate corrections (Johnson & Seifert, 1994). Thus, much research effort has focused on the conditions that promote effective updating of the situation model (for reviews see Chan et al., 2017; Lewandowsky et al., 2012; Seifert, 2002; Walter & Tukachinsy, 2020). A multitude of factors have been explored, including the timing of correction (e.g., Cook et al., 2017; Ecker et al., 2010; Ithisuphalap et al., 2020), prior encounters of misinformation (e.g., ; Ecker et al., 2017 ; Pennycook et al., 2018), prior beliefs (e.g., Ecker & Ang, 2019; Swire et al., 2017a, b; Swire-Thompson et al., 2020), and individual differences (e.g., Chang et al., 2019; Pennycook & Rand, 2019). Here, we focus on the content of the correction and the importance of the misinformation to the unfolding narrative.

Consistent with the situation model literature, multiple CIE studies have reported that presenting an alternative account (e.g., arson as a cause of the warehouse fire) to replace the discredited misinformation (e.g., the inference that the fire was caused by volatile materials in a nearby closet) can be an effective means of reducing the CIE (e.g., Ecker et al., 2010; Ecker et al., 2011a, b; Johnson & Seifert, 1994; Lewandowsky et al., 2012; Rich & Zaragoza, 2016, 2020; see Chan et al., 2017 for a review). For example, Johnson and Seifert (1994) reported that individuals who received both a correction statement and alternative information were less influenced by misinformation than those who received only a correction statement. Importantly, the participants from the first group performed similarly to those in a baseline condition who never heard the misinformation. In other words, individuals who received the correction and the alternative account were successful in fully updating their mental models, as though the misinformation was never encountered.

Although recent meta-analyses (Chan et al., 2017; Walter & Tukachinsy, 2020) corroborated that incorporating an alternative into the correction statement is an effective strategy in reducing the CIE, elimination of the CIE as reported by Johnson and Seifert (1994) appears to be more of an exception than the norm^1^ (Ecker et al., 2010; Lewandowsky et al., 2012; Lewandowsky et al., 2017; see Chan et al., 2017 for a review). For example, Ecker et al. (2010) found that the provision of an alternative account increases the effectiveness of the correction, but the CIE persisted, such that participants still referenced the misinformation when responding to inference questions (see also Ecker et al., 2011a, b; Rich & Zaragoza, 2016, 2020). Interpreting these results in the context of the situation model, when the initial misinformation is tagged as invalid, it leaves a narrative void. When an alternative is offered, it can fill the gap, and the revision completes the mental model and restores narrative coherence. When no alternative is available to fill the void, however, the event model is rendered incomplete. It has been suggested that readers would rather tolerate an inconsistent model that contains invalidated information than accept an incoherent model that contains a gap (e.g., Ecker et al., 2011a, b; Hamby et al., 2020). Thus, the influence of misinformation persists.

Other models suggest that even after a piece of information is discredited, it remains accessible from memory. In fact, the memory trace for the misinformation may linger, and when reactivated (by virtue of its prior associations), it will compete with the newly encoded alternative information (e.g., Ayers & Reder, 1998; Ecker et al., 2011a, b; Gordon et al., 2019; Kendeou & O’Brien, 2014). As the newly acquired alternative information builds its activation strength (e.g., by bolstering its connection with other knowledge units within the network), it may more successfully inhibit the previously discredited misinformation (Kendeou & O’Brien, 2014). In sum, although the activation level of the invalidated misinformation can be reduced and inhibited by competing alternative information, it can never be fully displaced. As such, the CIE may reflect instances where the activation level of the misinformation exceeds that of the alternative information.

One factor that likely determines initial and residual activation level of discredited misinformation is the idea unit’s importance in the narrative. By virtue of the central role important information plays in a narrative, more important (central) information will likely have greater inter-connections with other story details than less important (peripheral) information (Kendeou et al., 2019). Consequently, the higher activation level of central misinformation may provide stronger competition against the alternative information, thereby rendering it more resistant to correction than peripheral misinformation. Consistent with this notion, Wilkes and Leatherbarrow (1988) reported lower CIE for peripheral misinformation than central misinformation. We will return to these ideas later in the manuscript.

Another factor that has been linked to CIE mitigation is directness of the correction. One way to operationalize “directness” is whether the misinformation is targeted in the context of the correction statement. For example, Wilkes and Leatherbarrow (1988) included two conditions that differed in directness. In the direct editing condition, the misinformation was restated within the correction statement (e.g., “… no storage of inflammable materials had occurred / and the side room had been empty before the fire”). In the indirect editing condition, the correction statement only referenced the need for a correction but not its locus (e.g., “… stating that the earlier message was incorrect./The side room had been empty before the fire”). Although Wilkes and Leatherbarrow did not observe a difference between the two types of corrections, more recent evidence from Ecker et al. (2017) demonstrated that corrections that explicitly targeted the misinformation (by explicitly repeating it) were more effective in CIE reduction than those that did not. This pattern is consistent with the models described earlier (e.g., Ecker et al., 2011a, b; Johnson-Laird, 2012; Verschueren, Schaeken, & d’Ydewalle, 2005), where direct editing should be more effective than indirect editing as it targets a specific information unit that needs to be tagged and/or replaced rather than simply providing a generic statement that a correction is needed. In light of these mixed findings, it is worthwhile to further define the potential contribution of directness to CIE mitigation.

To summarize, we have discussed several factors that can effectively reduce the continued influence of misinformation. While these findings are consistent with the models we described earlier, their relative contribution toward CIE reduction remains unclear. Given the negative consequences associated with the reliance of misinformation, it is crucial to identify whether combining multiple strategies could enhance the effectiveness of corrections and retractions. Furthermore, as discussed above, given the scarcity of demonstrations of successful CIE elimination, it is important to revisit what correction strategy may be the most effective in neutralizing the continued influence of misinformation.

The studies reported here are designed to address these gaps in the literature. In Experiments 1A and 1B, we examined the effectiveness of correction statements that systematically combine alternative provision and directness of misinformation targeting. Importantly, by including two baseline conditions, we are able to directly test for potential CIE elimination and correction effectiveness (see Methods section for additional details about the baseline conditions). In Experiment 2, we further examined whether the combination of strategies may affect different types of misinformation in distinct ways. Together, these findings would contribute to our understanding of the persistence of misinformation.

## Method overview

In this section, we present the overall procedure used in all three studies. Experiment-specific methodology will be detailed in the respective sections.

### Design

We employed a between-subjects design, where individuals were randomly assigned to each condition.

### Procedure

After providing informed consent, participants were presented with the instructions to read a series of Twitter-style messages about a particular event (e.g., minibus accident in Experiment 1A). They were asked to read the story carefully because they would be asked some questions about it at the end. To ensure that participants understood the instructions, they were required to answer a multiple-choice question about the instructions. Individuals who correctly answered that question would proceed to the story, and those who failed the instruction check were presented with the instructions again and were then required to answer a different instruction check question. Selecting the correct answer would allow the individual to proceed, and failure to respond correctly would result in disqualification from the study.

**Fig. 1:**
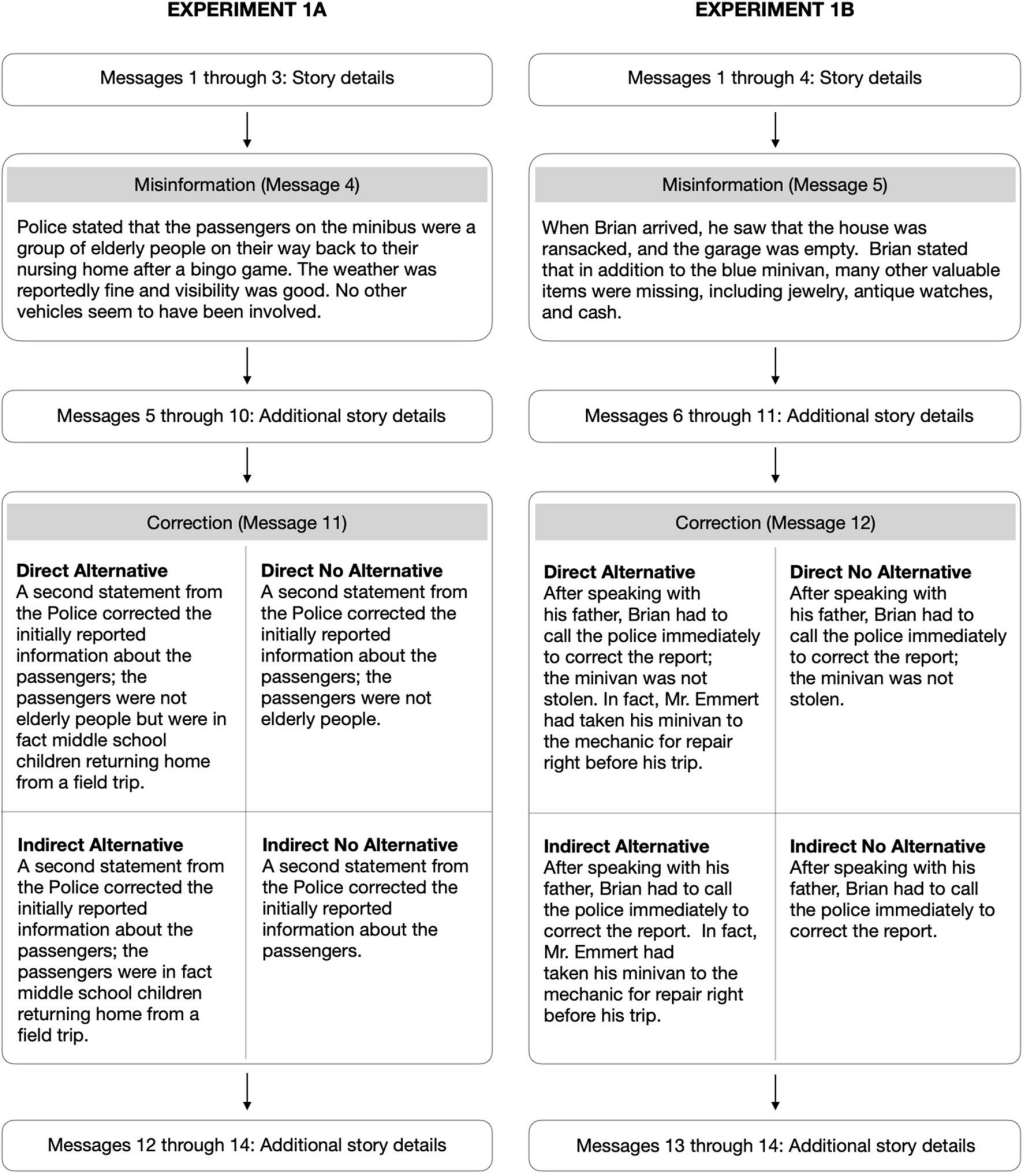
Summary of message sequence and additional details for experimental conditions in Experiment 1A (left panel) and Experiment 1B (right panel)

**Story presentation.** Consistent with other CIE studies (e.g., Johnson & Seifert, 1994; Wilkes & Leatherbarrow, 1988), each story was presented as a series of short messages, one at a time, and participants were not allowed to backtrack. The critical information was introduced toward the beginning of the narrative and the correction statement (when relevant) was presented toward the end of the story (see Figs. 1 and 5 for an overview). For the sake of simplicity, we will use the collective term (mis)information to refer to the critical information that would later be corrected for experimental subjects, would be left uncorrected for no correction baseline subjects, and would not be presented to no (mis)information baseline participants.

**Fig. 2:**
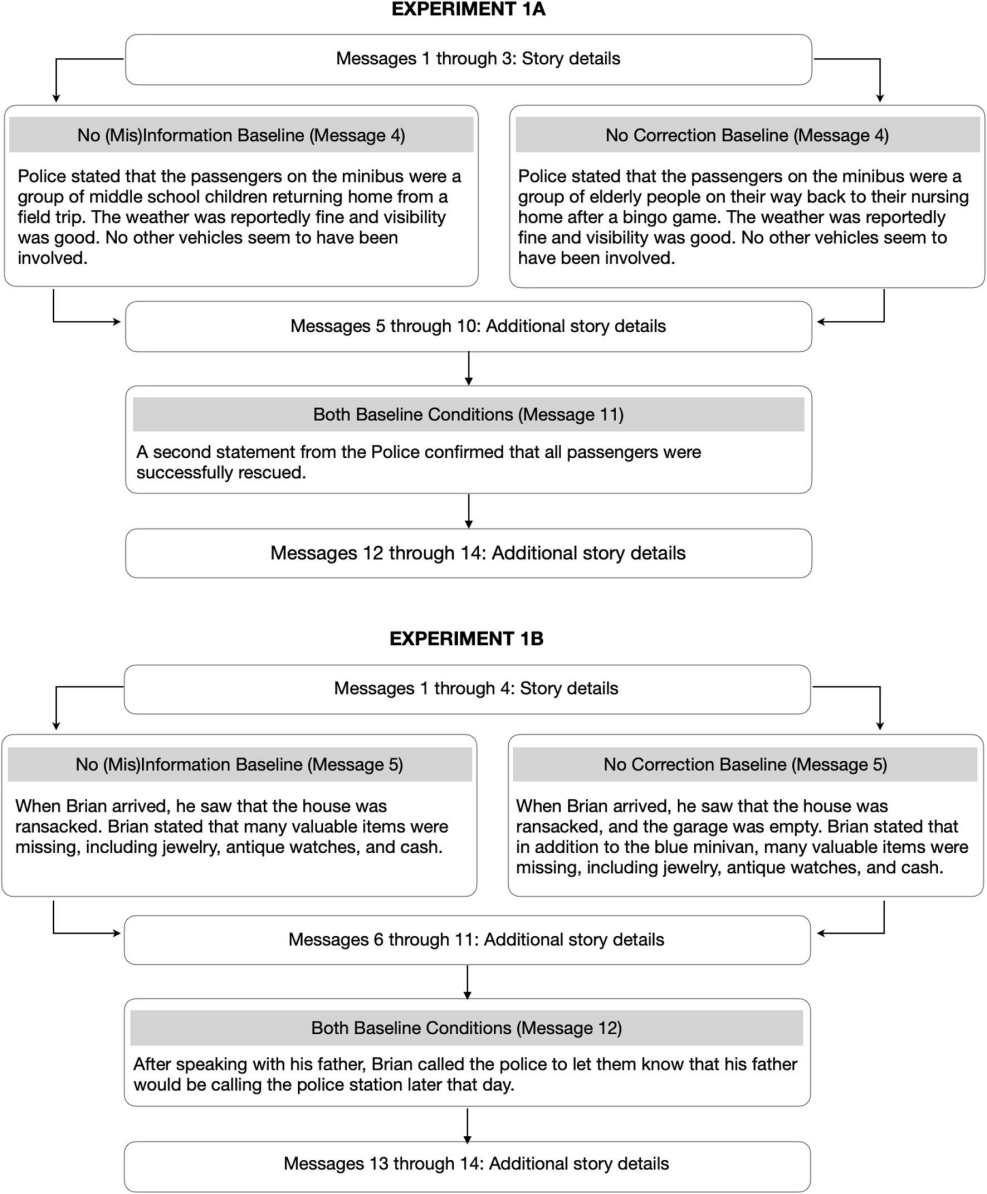
Summary of message sequence and additional details for baseline conditions in Experiment 1A (top) and Experiment 1B (bottom)

**Baseline conditions.** Each experiment included two baseline conditions (see Figs. 2 and 6 for an overview). Participants in the no correction baseline condition read the same critical message as individuals in the experimental conditions. Those in the no (mis)information baseline condition read a message that did not include the (mis)information. Instead, the no (mis)information baseline message presented a different set of facts about the event. Importantly, none of the baseline participants encountered a correction statement; instead, they read a filler statement. Thus, equivalent performance between no (mis)information baseline and experimental groups would suggest that the correction statements are highly effective, such that the experimental subjects would be behaving as though they had never encountered the misinformation. In contrast, equivalent performance between the no correction baseline and experimental groups would suggest that the correction statements are ineffective. That is, individuals in the experimental groups would behave as though they had never encountered the correction. In sum, comparisons between the no (mis)information baseline and the experimental conditions enabled us to assess the potential for CIE elimination, and contrasts between the no correction baseline and the experimental conditions allowed us to evaluate correction effectiveness. As noted earlier, since the no (mis)information baseline condition is often missing in other studies (for recent exceptions, see Connor Desai & Reimers, 2019; Ecker et al., 2020a, 2020b), our dual baseline approach may provide valuable insights regarding continued reliance on misinformation and success in mental model updating.

**Demographic questions and filler task.** After reading the story, individuals answered two demographic questions (age, gender) and completed an unrelated non-mnemonic filler task that lasted approximately 10 min.

**Probe questions.** Two types of probe questions were presented next: story-specific CIE questions and story comprehension questions (see Appendix B).

***CIE probe questions***. For each story, six questions were used to assess the CIE. Manner of assessment differed between Experiments 1 and 2, and details will be provided in the respective sections.

***Story comprehension questions.*** Several multiple-choice items were included to assess subjects’ comprehension. While all items assessed memory for story details (i.e., general comprehension questions), one item inquired about the (mis)information specifically. Thus, the general items served as a comprehension check. Data from individuals who scored below 50% on the general comprehension questions were excluded from further analyses, as that level of performance likely reflects poor comprehension. Performance was generally high, and only a small percentage of individuals in each experiment were excluded for poor performance (1.6% in Experiment 1A, 1.5% in Experiment 1B, and 2.6% in Experiment 2). The (mis)information-specific question provides a direct assessment of continued influence of invalidated information (for experimental groups), correct retention of that content (for no correction baseline participants), and spontaneous endorsement of unpresented information (for no (mis)information baseline subjects).

**Analyses.** Across experiments, we first focused on the CIE for experimental participants. We then conducted contrast analyses that compared each experimental condition against the no (mis)information baseline condition to evaluate potential CIE elimination and against the no correction baseline condition to assess correction strategy effectiveness. The Bonferroni correction for multiple comparisons was applied to all contrast analyses. When relevant, we also conducted equivalence tests (Lakens, 2017) to address limitations associated with null hypothesis testing. Additional analyses were included for Experiment 2, which will be described later.

**Summary.** All studies were hosted on Qualtrics (2019), an online data collection platform commonly used in behavioral research. After providing informed consent, participants completed the following tasks: (1) presentation of news story, (2) demographic questions and filler task, and (3) probe questions aimed to assess CIE and overall story comprehension.

## Experiment 1A

### Method

**Subjects.** Two hundred and fifty-two Villanova University students (*M* age = 18.9 years, SD = 0.9, range 18–23 years) participated for course credit. Subjects were randomly assigned to one of six conditions, with four conditions representing the 2 (alternative) × 2 (directness) factorial combination and two baseline conditions (no correction baseline and no (mis)information baseline). They were tested in small groups in a classroom setting, with empty seats around each person to minimize potential distraction. Across all conditions, four individuals’ data were excluded from subsequent analyses due to poor performance on the comprehension questions (< 50% correct). Thus, a total of 248 individuals provided usable data. All participants were native English speakers. Table 1 summarizes the characteristics of our sample.

**Table 1:** Number and Gender Distribution (female/male/do not wish to say) of Participants in Each Condition of Experiment 1A and Experiment 1B

	Experiment 1A (minibus)		Experiment 1B (burglary)	
	Alternative	No alternative	Alternative	No alternative
Direct	45 (35/10/0)	42 (24/18/0)	41 (33/8/0)	43 (29/13/1)
Indirect	41 (31/10/0)	45 (26/19/0)	41 (21/20/0)	47 (39/8/0)

Experiment 1A: 45 (25/20/0) individuals participated in the No (mis)information baseline condition and 30 (18/12/0) participated in the No correction baseline condition

Experiment 1B: 43 (36/7/0) individuals participated in the No (mis)information baseline condition and 43 (19/24/0) participated in the No correction baseline condition

Although a power analysis based on the mean effect sizes reported in Chan et al.’s meta-analysis, (2017) suggested that a minimum of 15 participants per condition would be sufficient to detect the effects of interest (assuming a 0.80 level of power, G*Power, UCLA: Statistical Consulting Group (2020)), we opted for a larger sample size to remain comparable with recent studies that used a similar paradigm (e.g., Connor Desai & Reimers, 2019; Ecker & Antonio, 2021, for a more recent meta-analysis where a wide range of sample sizes were reported, see Walter & Tukachinsky, 2020).

**Stimuli.** We adapted the minibus accident story used by Ecker et al. (2010). We manipulated the provision of an alternative account and the directness of the correction statement (see Fig. 1). We operationalized directness as whether the misinformation was directly referenced in the correction statement. See Figs. 1 and 2 for condition details and Appendix A for the entire story.

**Probe questions.** Six open-ended questions were included to assess CIE and five multiple choice items were posed to assess story comprehension. Among the comprehension questions, one directly inquired about the (mis)information (i.e., age of passenger) and the remaining four pertained to other story details.

## Experiment 1A Results

### Scoring of probe questions

**Open-ended (mis)information questions.** Two primary coders, blind to condition, scored all responses to the open-ended (mis)information probe questions. A third coder, also blind to condition, scored only the items that required a tie-breaker vote. Prior to data coding, a randomly selected set of responses from 15 participants were used as training material. All coders scored these responses, and all responses were compared and discussed. Consistent with previous CIE studies (e.g., Wilkes & Leatherbarrow, 1988), coders identified references made to the (mis)information in each response, which took the form of direct reference (e.g., “because they are old”) or thematic inference (e.g., “because they are frail,” which is consistent with the elderly stereotype). A response that referenced the (mis)information (i.e., elderly) received a score of 1, and a maximum of 1 point was assigned to each question, regardless of the number of references to the (mis)information. Furthermore, a response that did not reference the (mis)information received a score of 0. Similarly, ambiguous responses (e.g., “Because of their age”) or mixed responses that included both (mis)information and corrected alternative information (e.g., “Passengers were both elderly and young children.”) received a score of “0.” In other words, only uncontroverted references to the (mis)information contributed toward the total each participant’s (mis)information score (maximum six points).

Coder agreement was calculated for each (mis)information probe question after the initial round of coding. Agreement level was very high, with the two coders agreeing on 99.5% of all responses (question-level agreement ranged from 99.54 to 100%). For discrepant cases, the third coder’s scoring was used as a tie-breaker.

The identical scoring procedure was used for responses from the baseline conditions. The only difference lies in the interpretation. Rather than interpreting the (mis)information score as reflecting continued influence of misinformation, it instead is interpreted as reflecting retention of presented information (no correction baseline condition) or spontaneous reference to unpresented information (no (mis)information baseline condition).

**Comprehension questions.** Scoring of all forced choice questions was straightforward. For the question that was specific to the (mis)information, (i.e., “How old were the passengers?”), selection of the “elderly” response option by the experimental participants would be indicative of the persistence of misinformation.

### Analyses and results

**Fig. 3:**
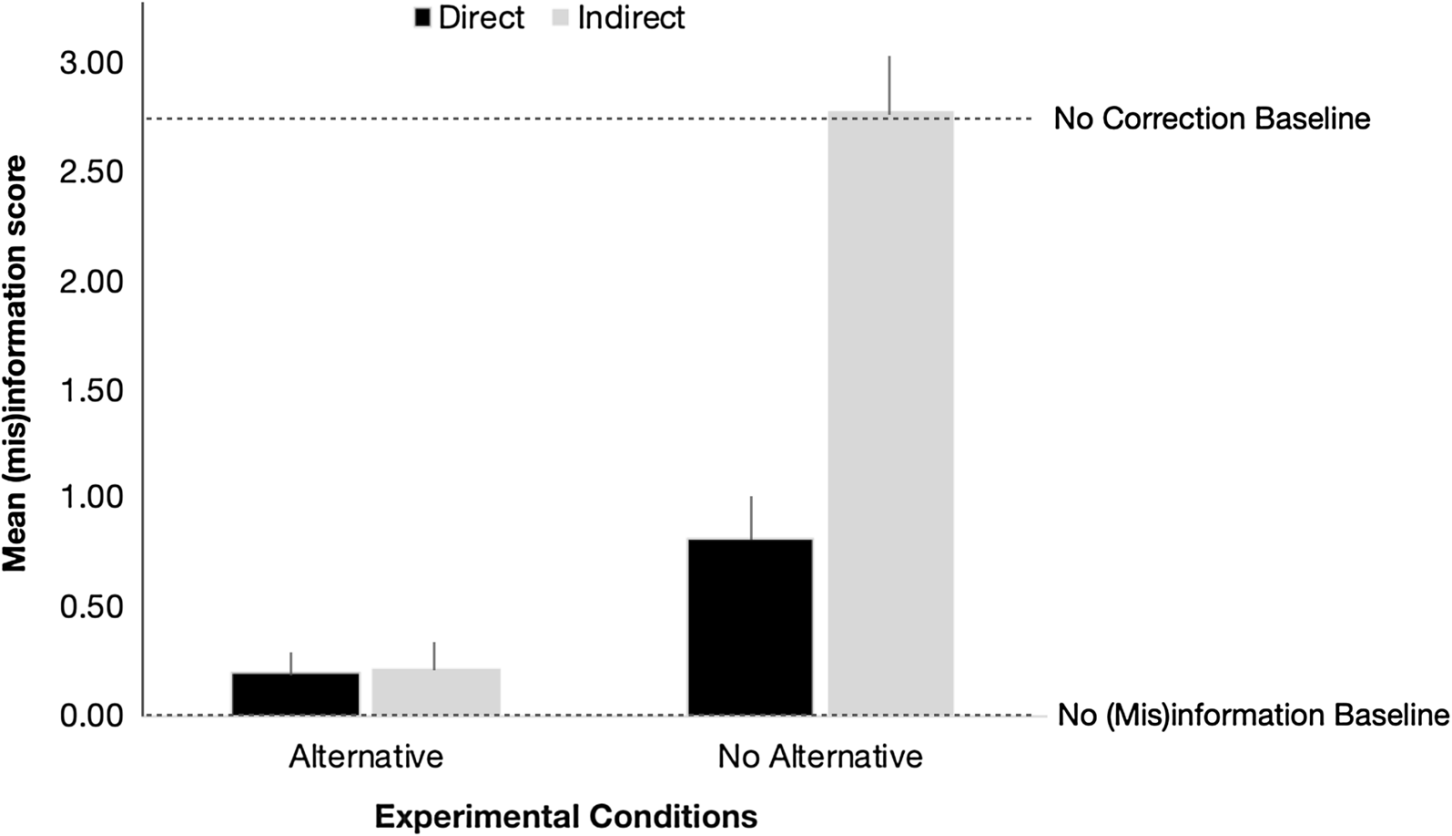
Mean (mis)information score in Experiment 1A. Means for baseline participants are indexed with dotted lines. Error bars indicate standard error of the mean

**Continued influence effect.** Figure 3 presents the mean (mis)information score across participants for each condition, with lower scores indicating fewer references to the (mis)information. We conducted a 2 (alternative) × 2 (directness) analysis of variance (ANOVA) on (mis)information score for the experimental subjects to examine the effects of alternative provision and correction directness. We found a significant 2-way interaction, *F*(1, 169) = 26.193, *p* < .001, *η*^2^_p_ = .13.

Probing of the simple effects revealed the following patterns. (a) When an alternative was included in the correction, directness of misinformation targeting did not appear to affect the (mis)information score, a pattern corroborated by an independent samples t test (direct alternative *M* = 0.20, SD = 0.66 vs. indirect alternative *M* = 0.22, SD = 0.82, *t*(84) =  − 0.122, *p* = .903, *η*^2^ = .00). However, a limitation of null hypothesis testing is that failure to find significant differences is not statistically equal to acceptance of the null hypothesis (Lakens, 2017). Therefore, we sought to verify equivalence by conducting an equivalence test, with the TOST procedure, which requires two one-sided t tests to determine whether the observed data points are within equivalence bounds. If both of these one-sided t tests are significant, equivalence is confirmed (see Lakens et al., 2018 for a review and tutorial of the procedure). For the purpose of reporting, only the one-sided test with the smaller t-statistic (i.e., larger *p* value) will be reported.

Returning to the data, the equivalence test confirmed that the (mis)information scores between the two conditions are equivalent, *t*(79) = 2.31, *p* = .012, confirming that when an alternative was provided, direct targeting of the misinformation did not further reduce CIE. (b) When the correction does not include an alternative, however, directly targeting the discredited information in the correction reduces the (mis)information score, such that the score was lower in the direct no alternative condition (*M* = 0.81, SD = 1.35) than in the indirect no alternative condition (*M* = 2.78, *SD* = 1.81), *t*(85) =  − 5.725, *p* < .001, *η*^2^ = .28. (c) When the invalidated information was directly targeted in the correction statement, inclusion of an alternative account significantly decreased the (mis)information score compared to when an alternative account is not provided, *t*(85) =  − 2.706, *p* = .008, *η*^2^ = .08. (d) This pattern was also observed when the discredited information was not directly targeted in the correction statement, *t*(84) =  − 8.310, *p* < .001, *η*^2^ = .45. Thus, the significant interaction suggests that the benefit of alternative provision was more apparent when the correction does not directly target the misinformation.

**Testing for potential elimination of continued influence.** Although the analyses above demonstrated varying degrees of CIE mitigation, they did not address whether any of the correction strategies succeeded in eliminating the CIE. To evaluate this, we conducted hypothesis-driven contrast analyses to identify conditions that would warrant equivalence testing (summarized in Table 2). Specifically, we contrasted the mean number of references to the elderly in each experimental condition against the no (mis)information baseline condition (Ecker et al., 2010). To reiterate, similar behavior between the no (mis)information baseline and experimental groups would suggest that the experimental participants behaved as though they never encountered the misinformation.

**Table 2:** Summary of Contrast Analyses to Test for CIE Elimination in Experiment 1A. Contrast Values Refer to Difference in Mean (Mis)information Score Between an Experimental Condition and the No (Mis)information Baseline Condition

Contrast	Direct alternative	Direct no alternative	Indirect alternative	Indirect no alternative	No (mis)information baseline	Contrast value	*t(198), p *value, eta^2^
1	1	0	0	0	− 1	0.20	0.851, .396, .00
2	0	1	0	0	− 1	0.81	3.386, .001, .06
3	0	0	1	0	− 1	0.22	0.912, .363, .00
4	0	0	0	1	− 1	2.78	11.825, < .001, .41

One-way ANOVA, *F*(4, 213) = 47.277, *p* < .001

As summarized in Table 2, two contrasts failed to reach significance using null hypothesis testing. However, equivalence testing showed that neither comparison was statistically equivalent (direct alternative vs. no (mis)information baseline, *t*(44) =  − .48, *p* = .315; indirect alternative vs. no (mis)information baseline, *t*(40) =  − .69, *p* = .248), suggesting that neither strategy eliminated the CIE.

**Testing for correction effectiveness.** Table 3 summarizes the contrast analyses that compared performance between experimental and no correction baseline participants. The only comparison that failed to reach significance using null hypothesis testing was that between the indirect no alternative and no correction baseline conditions. Equivalence testing confirmed that these conditions were equivalent, *t*(84) =  − 2.33, *p* = .011, verifying that although participants in the indirect no alternative condition received a correction statement, they made as many references to the (mis)information as individuals who never encountered a correction. Thus, we conclude that a correction statement is ineffectual if it only made vague reference to the misinformation and failed to include an alternative.

**Table 3:** Summary of Contrast Analyses to Test for Correction Effectiveness in Experiment 1A. Contrast Value Refers to the Difference in Mean (Mis)information Score Between an Experimental Condition and the No Correction Baseline Condition

Contrast	Direct alternative	Direct no alternative	Indirect alternative	Indirect no alternative	No correction baseline	Contrast value	*t(198), p *value, eta^2^
1	− 1	0	0	0	1	2.53	7.967, < .001, .24
2	0	− 1	0	0	1	1.92	5.966, < .001, .15
3	0	0	− 1	0	1	2.51	7.756, < .001, .23
4	0	0	0	− 1	1	− 0.04	− 0.140, .889, .00

One-way ANOVA, *F*(4, 198) = 37.393, *p* < .001

**Explicit endorsement of misinformation.** We also included a single multiple-choice question that asked about the age of the passengers, with the options being young, middle-aged, or elderly. For experimental subjects, endorsement of “elderly” indicated a continued reliance on invalidated information. For no (mis)information baseline participants, selection of “elderly” was an error, and for no correction baseline subjects, endorsing “elderly” was the correct answer. We calculated the percentage of participants in each condition who endorsed the “elderly” option: direct alternative = 6.7%, direct no alternative = 38.1%, indirect alternative = 4.9%, indirect no alternative = 95.6%, no (mis)information baseline = 2.2%, and no correction baseline = 96.7%. These patterns mirrored those observed with open-ended CIE probe questions (see Fig. 3).

**Comprehension questions.** Overall, participants performed quite well on the comprehension questions, with a mean percent accuracy of 79.4% (*SD* = 15.8%), suggesting that they engaged with the information in a meaningful manner.

## Experiment 1A Discussion

In Experiment 1A, we evaluated the extent to which correction statements that systematically combine alternative provision and directness of misinformation targeting could reduce the CIE. Furthermore, by including two baseline conditions, we were able to assess potential CIE elimination and correction effectiveness.

We found that both factors contributed to CIE mitigation, and these factors appear to work in conjunction. Specifically, (mis)information scores were lower when an alternative was provided than when it was not, and this benefit was larger for the indirect condition than the direct condition. Furthermore, we observed that participants who read the indirect no alternative correction statement made as many references to the (mis)information as those who never received a correction, suggesting that the correction strategy was wholly ineffectual.

Based on the literature reviewed above, one might expect the direct alternative condition to be the most effective in CIE reduction because it combines two factors that have previously been demonstrated to successfully lower CIE. However, we did not observe such a “super correction” effect. A closer examination of the indirect alternative correction statement may provide some insight. The indirect alternative correction statement read, “A second statement from the Police corrected the initially reported information about the passengers; the passengers were in fact middle school children returning home from a field trip.” Even though the correction did not directly reference the misinformation (i.e., elderly passengers), the alternative account in the correction statement (i.e., “…in fact middle school children”) may have included sufficient detail to pinpoint what unit of information (i.e., age of passengers) should be updated, thereby unintentionally equalizing the direct alternative and indirect alternative conditions. Finally, none of these correction strategies completely eliminated the CIE, a pattern that is consistent with the vast literature that has demonstrated the robustness of this effect (for reviews, Lewandowsky et al., 2012; Rapp & Salovich, 2018; Seifert, 2002; for meta-analyses, see Chan et al., 2017; Walter & Tukachinsky, 2020). The broader implications of these findings will be discussed in the General Discussion.

## Experiment 1B

The primary goal of Experiment 1B was to introduce an internal replication of the paradigm used in Experiment 1A with a different story (home burglary). When constructing the new story, we also addressed the possible unintended equalization of the direct alternative and indirect alternative correction statements in Experiment 1A. Here, the correction statement in the indirect alternative condition alluded to the misinformation (minivan) but did not specifically reference the theft of the vehicle (see Fig. 1).

## Method

**Subjects.** Two hundred and sixty-two Villanova University students (*M* age = 19.7 years, SD = 2.0, range 18—37 years) participated for a chance to enter into a raffle drawing. Participants were randomly assigned to one of six conditions, with four conditions representing the 2 (alternative) × 2 (directness) factorial combination and two baseline conditions. All individuals participated remotely, at a quiet setting of their own choosing. Across all conditions, four individuals’ data were excluded from subsequent analyses due to poor performance on the general comprehension questions (< 50% correct). Thus, a total of 258 individuals yielded usable data. All participants are native English speakers. See Table 1 for demographic characteristics for our sample.

**Stimuli.** We constructed a burglary story that is based loosely on the jewelry theft story from Johnson and Seifert (1994). See Figs. 1 and 2 for a summary of the conditions and Appendix A for the complete story.

**Probe questions.** The types of probe questions included are identical to those in Experiment 1A. The only difference is that a total of six comprehension questions (one directly assessed CIE and five inquired about other details) were used in Experiment 1B. See Appendix B for the list of questions.

**Procedure.** All procedures are identical to Experiment 1A.

## Experiment 1B Results

### Scoring of probe questions

We employed the same scoring procedures outlined in Experiment 1A. Across all six questions, the two coders agreed on 98.7% of all responses (question-level agreement ranged from 97.1 to 100%).

### Analyses and results

**Fig. 4:**
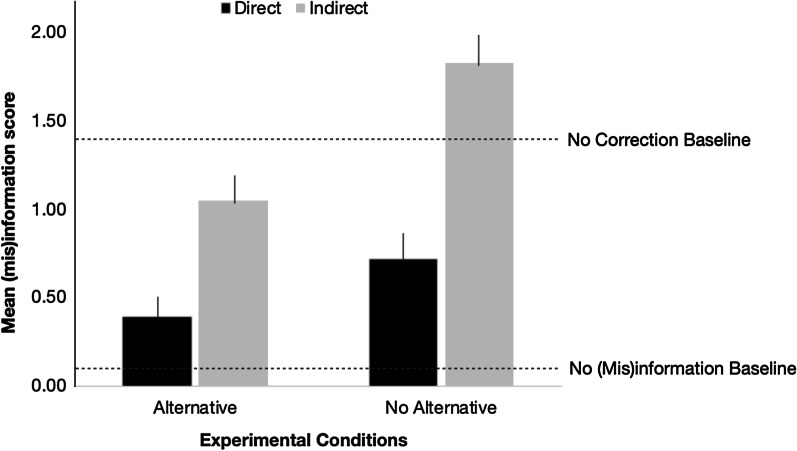
Mean (mis)information score in Experiment 1B. Means for baseline participants are indexed with dotted lines. Error bars indicate standard error of the mean

**Continued influence effect.** For each participant, we calculated a (mis)information score by tabulating the number of trials in which participants unambiguously referenced “minivan” in their open-ended responses. Figure 4 presents the means across participants and conditions, with lower values indicating fewer references to the (mis)information.

To examine the effects of alternative provision and correction directness on CIE, we conducted a 2 (alternative) × 2 (directness) ANOVA on (mis)information score. We found a significant alternative main effect, *F*(1, 168) = 13.414, *p* < .001, *η*^2^_p_ = .07, suggesting that the CIE was reduced when an alternative was available (*M* = 0.72, SD = 0.91) compared to when an alternative was unavailable (*M* = 1.29, SD = 1.20). We also observed a significant directness main effect, *F*(1, 168) = 34.397, *p* < .001, *η*^2^_p_ = .17, indicating that the CIE was reduced when the misinformation was directly targeted in the correction (*M* = 0.56, SD = 0.87) compared to when the misinformation was indirectly targeted in the correction (*M* = 1.45, SD = 1.13). However, the interaction between alternative and directness failed to reach significance, *F*(1, 168) = 2.077, *p* = .151, η^2^_p_ = .01, suggesting that these two factors operated independently.

**Testing for potential elimination of continued influence.** As in Experiment 1A, we asked whether the CIE was eliminated in any of our experimental conditions by taking a contrast analysis approach. Before discussing the analysis, it is interesting to note that although participants in the no (mis)information baseline condition never read about a stolen minivan, some of them made reference to it. Such intrusions may reflect the fact that the unpresented idea is plausible within one’s schema of “home burglary.” Indeed, this pattern is consistent with the vast literature on schema-driven memory errors (e.g., for classic examples, see Bartlett, 1932; Brewer & Treyens, 1981). Importantly, such spontaneous references do not impact our rationale for the contrast analyses.

Instead of using the (mis)information score in this analysis, as we did in Experiment 1A, we utilized a (mis)information percentage score instead. This was because we excluded one of the CIE probe questions (“Where would Mr. Emmert go to pick up his minivan when he returned?”) from the no (mis)information baseline condition, as the question would appear nonsensical to those participants who had not encountered any information about a minivan in the story. To allow for comparison across conditions with a different number of trials, we calculated a (mis)information percentage score for each person, where we divided each (mis)information score by six for the experimental participants and by five for the no (mis)information baseline participants.

As shown in Table 4, only the contrast between the direct alternative and the no (mis)information baseline conditions failed to reach significance using null hypothesis testing. Importantly, the equivalence test confirms that we successfully eliminated the CIE—when the correction statement targets the misinformation and includes an alternative, participants performed the same as those who never heard the misinformation, *t*(48) =  − 1.81, *p* = .038.

**Table 4:** Contrast Analyses to Test for CIE Elimination in Experiment 1B. Contrast Values Refer to Difference in Mean (Mis)information Percentage Between an Experimental Condition and the No (Mis)information Baseline Condition

Contrast	Direct alternative	Direct no alternative	Indirect alternative	Indirect no alternative	No (mis)information baseline	Contrast value	*t(210), p *value, eta^2^
1	1	0	0	0	− 1	0.04	1.140, .256, .01
2	0	1	0	0	− 1	0.09	2.866, .005, .03
3	0	0	1	0	− 1	0.15	4.509, < .001, .09
4	0	0	0	1	− 1	0.28	8.797, < .001, .27

One-way ANOVA*, F*(4, 210) = 23.744, *p* < .001

**Testing for correction effectiveness.** Table 5 summarizes the contrast analyses. While two contrasts were not found to be significantly different using null hypothesis testing, equivalence tests revealed that these conditions were not statistically equivalent (indirect alternative vs. no correction baseline, *t*(82) = .58, *p* = .28; indirect no alternative vs. no correction baseline, *t*(88) =  − .83, *p* = .21).

**Table 5:** Contrast Analyses to Test for Correction Effectiveness in Experiment 1B. Contrast Values Refer to Difference in Mean (Mis)information Score Between an Experimental Condition and the No Correction Baseline Condition

Contrast	Direct alternative	Direct no alternative	Indirect alternative	Indirect no alternative	No correction baseline	Contrast value	*t(210), p *value, eta^2^
1	− 1	0	0	0	1	1.05	4.959, < .001, .10
2	0	− 1	0	0	1	0.72	3.441, .001, .05
3	0	0	− 1	0	1	0.39	1.854, .065, .02
4	0	0	0	− 1	1	− 0.37	− 1.788, .075, .01

*One-way ANOVA, F*(4, 210) = 14.717, *p* < .001

**Explicit endorsement of misinformation.** We also included a yes–no forced choice question that asked whether the minivan was stolen. For experimental subjects, a “yes” response indicated a continued reliance on discredited information. For no (mis)information baseline participants, selection of that option was an error, and for no correction baseline subjects, selecting “yes” would be the correct answer. We calculated the percentage of participants who answered “yes” in each condition: direct alternative = 2.4%, direct no alternative = 2.3%, indirect alternative = 14.6%, indirect no alternative = 66.0%, no (mis)information baseline = 2.2%, and no correction baseline = 96.7%. These patterns mirrored those from the open-ended CIE probe questions (see Fig. 4).

## Experiment 1B Discussion

Similar to Experiment 1A, we found that both alternative provision and directness of misinformation targeting in the correction statement affected individuals’ reliance on discredited information. In contrast to Experiment 1A, however, these two factors appeared to operate independently in the current experiment. We found a significant reduction in the CIE when an alternative account was provided compared to when it was not, and we also found that the CIE was lower when the correction statement directly targeted the misinformation compared to when it did not. Finally, we also eliminated the CIE in the direct alternative condition.

The different patterns of findings across the two studies may be explained by several factors. First, as described earlier, a limitation of Experiment 1A was that we might have unintentionally equalized the direct alternative and indirect alternative conditions. After addressing that issue in Experiment 1B, the previously observed interaction effect was no longer apparent.

Second, the granularity of the misinformation may have differed between the two stories. The age of the passengers is arguably a self-contained idea unit, whereas the minivan is among one of the components of the idea unit of “stolen items” (among other components like cash and jewelry). Consequently, the same correction strategy may have a differential impact on these different types of information, where it may be easier to discount and update a standalone idea unit than a part of an idea unit. However, given the multitude of differences between the two stories, we are unable to directly assess this possibility.

Third, a comparison of the spontaneous references to the unpresented (mis)information between the two no (mis)information baseline groups may also be instructive in highlighting the differences between the stories. We first focused on the forced-choice questions that directly assessed the age of the passengers (Experiment 1A) and whether the minivan was stolen (Experiment 1B). Among the participants who never encountered the (mis)information, we found that one person indicated that the passengers were elderly (Experiment 1A), and 10 participants stated that the minivan was stolen (Experiment 1B). One possible interpretation of this pattern is that a stolen vehicle is highly associated with the gist of a “home burglary.” Thus, even though the (mis)information was never presented, individuals were willing to endorse this highly plausible event for the given context. In contrast, when the association was less strong, such as that between elderly passengers and minibus accident, there were far fewer spontaneous endorsements of the unpresented information. In other words, the strength of the associations between the (mis)information and the theme of the story might have played a role here.^2^

This explanation is consistent with the notion of “centrality” in the text comprehension literature, where centrality is commonly defined as the strength and/or number of conceptual connections an idea unit possesses. Relative to peripheral ideas, central ideas share stronger and/or more connections with other idea units within the narrative. (e.g., Miller & Keenan, 2011; Trabasso & Sperry, 1985; Yeari et al., 2017). Based on this definition, one may consider another difference between the two stories to be the centrality of the (mis)information: where stolen vehicle may represent a central idea for the home burglary story, and the age of the passenger may represent a peripheral idea for the minibus accident story.

Indeed, prior studies have demonstrated that centrality impacts correction effectiveness. For example, Wilkes and Leatherbarrow (1988) reported that it was easier to update the misinformation when it was peripheral to the narrative. This finding is consistent with the situation model framework, which suggests that text comprehension is critically dependent on narrative coherence. And when new information is introduced, coherence is temporarily disrupted while the model is updated, which involves either weaving the information into the mental model or by “outdating” the obsolete information. The “outdating” process involves two steps: discounting old information, and when available, replacing the gap left by the displaced information with newly introduced material (e.g., Kendeou et al., 2013; O’Brien et al., 2010; van Dijk & Kintsch, 1983). Returning to the idea of “centrality,” it is easier to outdate peripheral information because it has fewer and/or weaker connections to the rest of the story. When the to-be-replaced information is central, however, the disruption to narrative coherence is likely to be greater, and the corrections may be less likely to take hold, especially when there is no replacement information to fill the void (see Hamby et al., 2020 for related findings). It has been suggested that rather than accepting an incomplete mental model, readers would rather accept an inconsistent model that includes discredited information, thereby allowing the influence of misinformation to linger (e.g., Ecker et al., 2011a, b; Hamby et al., 2020). We examined the issue of centrality in Experiment 2.

## Experiment 2

The first goal of Experiment 2 was to investigate whether centrality of the misinformation affects correction effectiveness. Specifically, we asked: were the correction strategies introduced in Experiments 1A and 1B equally effective in correcting misinformation that was central to the narrative and misinformation that was peripheral to the narrative? We did so by systematically manipulating three factors—provision of an alternative, directness of misinformation targeting in the correction statement, and centrality of misinformation—in a single study. To our knowledge, this combination has not yet been examined in the literature.

In line with the text comprehension literature, we define centrality in terms of the narrative importance of the (mis)information to the rest of the story. One broad index of importance is whether the (mis)information plays a causal role in the narrative. By definition, causal information is central because it has important downstream consequences for the remainder of the story (e.g., Bower & Morrow, 1990; Morishima, 2016; Singer et al., 1992; van den Broek & Trabasso, 1986). Thus, we reason that causal information is likely to have more connections with the rest of the story than non-causal information. We manipulated centrality in the context of a story about a laundromat fire, where the misinformation pertained to either the cause of the fire (central) or the spread of the fire (peripheral).

In Experiment 1B, we found evidence for CIE elimination, which is relatively rare in the literature (Johnson & Seifert, 1994; for meta-analyses, see Chan et al., 2017; Walter & Tukachinsy, 2020). In addition to ensuring that this effect was reproducible with different stimuli, we also aimed to further delineate the component processes involved in “outdating” of misinformation in Experiment 2. Although successful outdating is assumed when participants no longer reference discredited misinformation, it can be difficult to confirm with the traditional CIE assessment utilizing open-ended questions.

First, open-ended responses can be quite idiosyncratic. For example, in response to the question, “Why do you think it was difficult getting both the injured and uninjured passengers out of the minibus?”, some answers were terse (e.g., “They were elderly”) while others were more comprehensive (e.g., “Because the uninjured passengers were also elderly and likely had various physical ailments or obstacles that prevented them from possessing a full range of motion.”). Since both of these responses made unambiguous references to the misinformation, they each received 1 point. However, this type of scoring procedure may obscure potentially relevant qualitative differences between the responses. Furthermore, it was not possible to ascertain whether these dissimilarities reflect differences in the underlying mental representations or simply individual differences in response style.

Relatedly, since our analysis in Experiments 1A and 1B only focused on incontrovertible CIE responses, we missed the opportunity to characterize other types of responses that could be theoretically interesting. Consider the following non-CIE responses from Experiment 1A:

1. “Because they were young and scared and there was a hill”
2. “Well, at first I thought it was difficult since they were elderly people, and it was difficult to get up after falling down, but since it actually was children, then it was because they must have been confused on what happened and had a difficult time understanding what to do.”
3. “The bus crashed on a steep embankment”

On the surface, all three responses indicate a non-reliance on discredited misinformation (i.e., non-CIE responses). However, closer examination of the three responses suggested that different processes may be at play. Although we assumed successful updating of the mental model in the first two responses, only the second response provided clear evidence of misinformation discounting *and* successful replacement with provided alternative information. Furthermore, it would be impossible to ascertain the details of the mental model in the third response as the respondent did not reference the passengers at all. In sum, these theoretically interesting differences may go unnoticed with the traditional CIE coding scheme, and this limitation is further exacerbated by the inherent idiosyncrasies of open-ended responses.

To circumvent these limitations, Experiment 2 used close-ended questions to assess CIE, a relatively novel approach in the literature (see Connor Desai & Reimers, 2019; Ecker et al., 2020a, 2020b). In a recent study, Connor Desai and Reimers (2019) found that both open- and close-ended questions readily elicited the CIE, and the patterns of responses were largely similar. Furthermore, they found that close-ended questions resulted in fewer dropouts, which was likely tied to reduced response burden.

Another advantage of the close-ended approach is that we were able to present respondents with both the misinformation and alternative information as response options. By allowing them to select more than one answer, we could infer whether (a) the misinformation persists and was the only active representation (i.e., endorsing *only* the misinformation option), (b) the misinformation lingered and competed with the provided alternative (i.e., endorsing both the misinformation and alternative options), or (c) the misinformation had been replaced by the provided alternative, thereby completing both parts of the outdating process (i.e., *not* endorsing the misinformation option *and* selecting the alternative option). Being able to tease apart these scenarios will contribute to our understanding of the component processes that contribute to the persistence of misinformation.

To summarize, we examined the effectiveness of correction strategies that reflect the factorial combination of alternative provision and correction directness on misinformation that was central and peripheral to the narrative. We did so using close-ended questions, which allowed us to further delineate the component processes involved in outdating misinformation.

## Experiment 2 Method

**Subjects.** One hundred and fifty-two native English speakers (*M* age = 19.9 years, SD = 1.7, range 18–28 years) participated for a chance to enter into a raffle drawing. Individuals were randomly assigned to one of 10 conditions, with eight conditions representing the 2 (alternative) × 2 (directness) × 2 (causality) factorial combination and two baseline conditions. All individuals participated remotely, at a quiet setting of their own choosing. Across all conditions, four individuals’ data were excluded from subsequent analyses due to poor performance on the general comprehension questions (< 50% correct). Thus, a total of 148 individuals yielded usable data. Table 6 summarizes the characteristics of our sample.

**Table 6:** Number and Gender Distribution (female/male/do not wish to say) of Participants in Each Condition of Experiment 2

	Causal		Non-causal	
	Alternative	No alternative	Alternative	No alternative
Direct	19 (15/4/0)	19 (14/5/0)	19 (16/3/0)	20 (15/4/1)
Indirect	17 (13/3/1)	18 (13/5/0)	18 (15/3/0)	18 (14/4/0)

24 (24/0/0) individuals participated in the No (mis)information baseline and 18 (14/4/0) individuals participated in the No correction baseline condition

Although a wide range of sample sizes have been utilized in the literature, we determined the current sample size based on the effect sizes from our own experimental manipulations in Experiments 1A and 1B. A power analysis suggested that a minimum of 13 individuals per condition would be sufficient to detect the effects of interest, assuming a .80 level of power (G*Power, UCLA: Statistical Consulting Group).

**Fig. 5:**
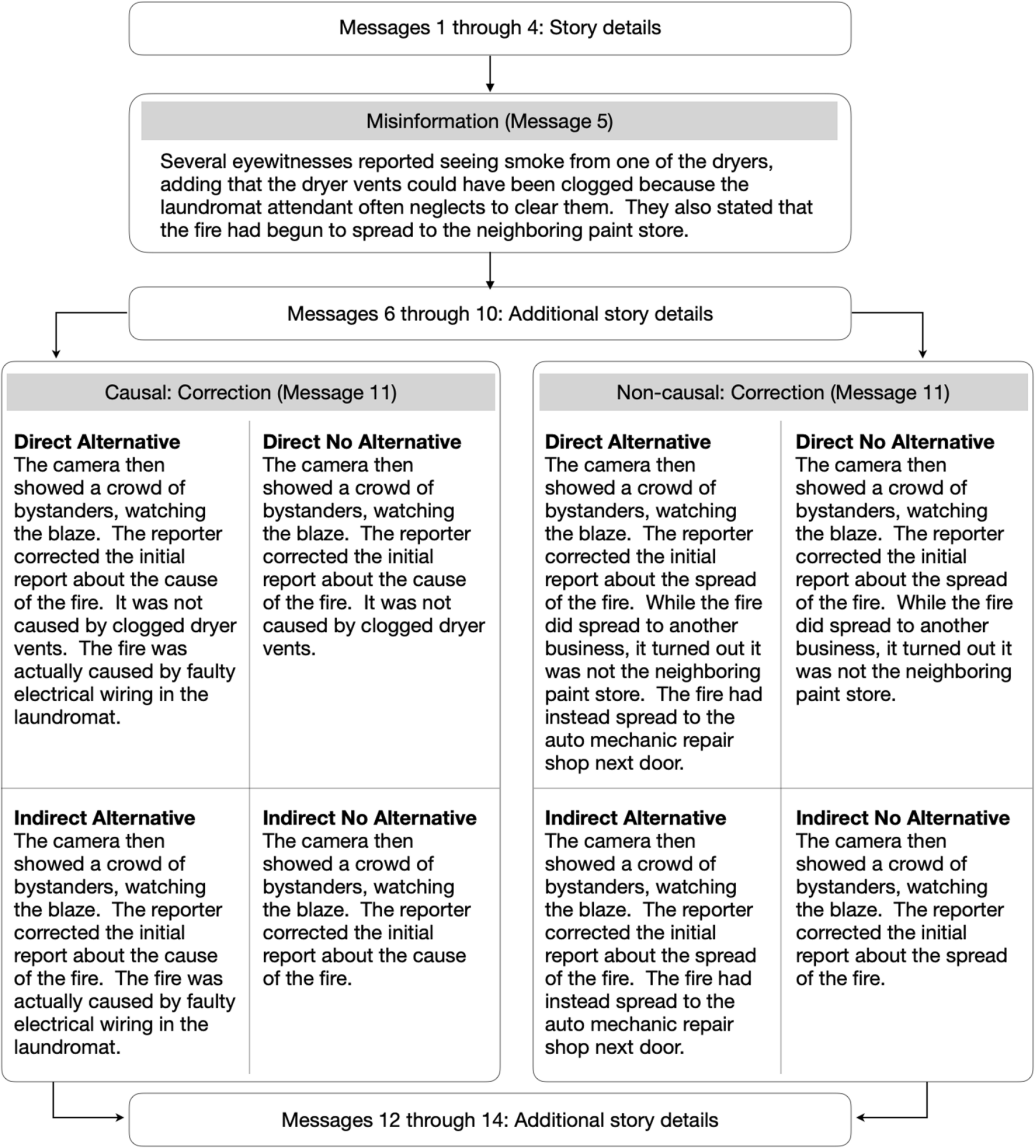
Summary of message sequence and story details for experimental conditions in Experiment 2

**Fig. 6:**
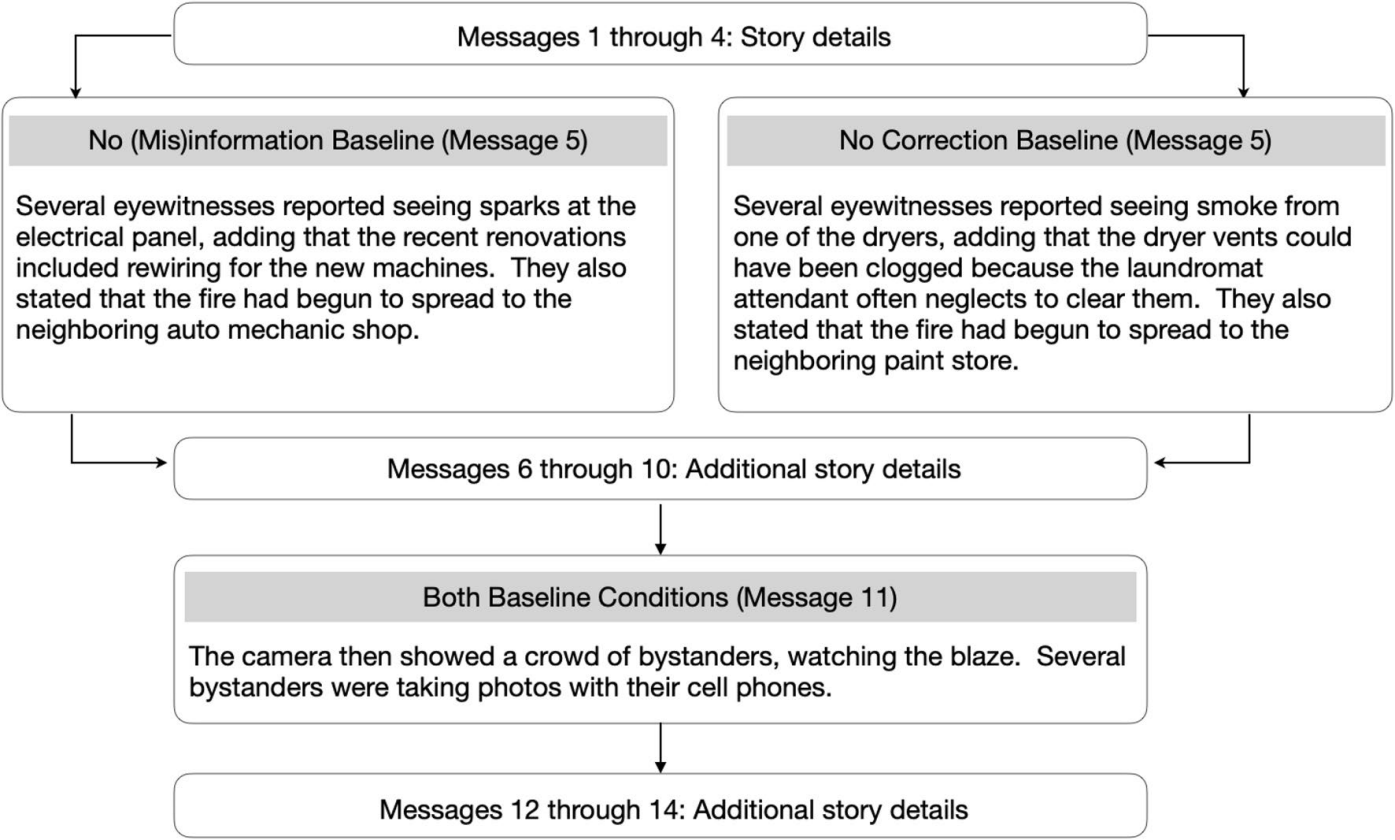
Summary of message sequence and additional details for baseline conditions in Experiment 2

**Stimuli.** We constructed a fictitious story about a laundromat fire. The critical message included information about both the cause and the spread of the fire. Importantly, all experimental participants encountered the identical critical message toward the beginning of the story. Depending on the condition, readers in the experimental groups would encounter a correction statement that concerned either the cause or the spread, and baseline participants read a filler statement. See Figs. 5 and 6 for summaries of the experimental and baseline conditions and Appendix A for the complete story.

**Probe questions.** We included six CIE questions and seven comprehension questions. Among the comprehension questions, one directly inquired about the causal (mis)information (i.e., cause of fire), one asked about the non-causal (mis)information (i.e., spread of fire), and the remaining five pertained to other details of the story. Question order within each type was randomized for each person. See Appendix B for the list of questions and response options.

For each CIE question, six response options were available, with two choices related to the cause of the fire, two choices related to the spread of the fire, one option about other story details, and a final “none of the above” option. The critical options were those about the cause and spread of the fire, whereas the other options were included to reduce the emphasis on cause and location and were not of primary interest. As such, data from those options were not included in the subsequent analyses and discussion. Response choices for each question, except “none of the above,” which was always presented as the final option, were also randomized across subjects.

Specifically, for each question, respondents in the causal alternative conditions encountered the following critical choices: misinformation (i.e., clogged vents), presented alternative (i.e., electrical wiring problems), presented non-causal information (i.e., paint store), and unpresented non-causal information (i.e., auto mechanic shop). Non-causal alternative participants viewed the same response options, but the corresponding classification differed: presented causal information (i.e., clogged vents), unpresented causal information (i.e., electrical wiring problems), misinformation (i.e., paint store), and presented alternative (i.e., auto mechanic shop). Selection of any unpresented options (which includes no alternative participants endorsing the alternative options) likely indicated guessing.

Among the comprehension questions, one item asked about the cause of the fire, and the other asked about the spread of the fire. Therefore, the question about the cause of the fire assessed CIE for causal subjects and general comprehension for the non-causal subjects. Similarly, the question about the spread of the fire represented a CIE question for the non-causal subjects and a general comprehension question for the causal subjects. Each comprehension question included three response options (see Appendix B).

Finally, participants were instructed to “select all that apply.” We reasoned that the combination of options would reveal representations that were active in the participants’ mental models at the time of responding. See Results section for additional details.

**Procedure.** All procedures are identical to Experiments 1A and 1B.

## Experiment 2 Results

### Scoring of probe questions

To streamline our results, we focus analyses and discussions on response selections that can be discernibly mapped onto different degrees of success in mental model updating: no update (i.e., CIE), partial update (i.e., competing representations or successful discounting but failed replacement), and full update (i.e., successful discounting and replacement). Thus, we only analyzed causal options for causal subjects and non-causal options for non-causal subjects. We explain below how each subject’s choice pattern for a single question — misinformation only, alternative only, both misinformation and alternative, or neither — mapped onto these different levels of updating.

**No update (i.e., CIE).** For all experimental participants, selecting only the misinformation option reflected continued reliance on discredited misinformation.

**Partial update: Competing representations.** For participants who received an alternative, selecting both the misinformation and the alternative options represented concurrent activation of both idea units.

**Partial update: Successful discounting but failure to replace with alternative.** For those who received an alternative, *not* selecting the misinformation option suggested successful discounting of discredited misinformation. In conjunction, *not* selecting the alternative option revealed that they failed to replace the narrative gap with the provided alternative.

**Partial update: Successful discounting when no alternative is available.** For participants who did not receive an alternative, we were only able to evaluate discounting success. Since they only encountered the misinformation, *not* selecting that option indicated successful discounting of invalidated information.

**Full update (i.e., complete outdating).** For individuals who received an alternative, *not* selecting the misinformation option confirmed successful discounting of misinformation. In conjunction, endorsement of the alternative option denoted successful replacement with the presented alternative. Thereby completing both steps of the outdating process.

**Other choice combinations.** Finally, we did not evaluate other choice combinations that did not clearly speak to the underlying mental model. For example, selection of any unpresented options (such as when participants who received no alternative endorsed the alternative option) likely reflected guessing at the time of testing rather than activation that resulted from memory retrieval.

For baseline participants, we examined both causal and non-causal responses, where causal responses served as baseline for the causal experimental groups, and non-causal responses served as baseline for the non-causal experimental groups. Specifically, we focused on instances when they endorsed the presented information and cases where they endorsed the unpresented information.

We followed this categorization procedure for each question for each participant. We then calculated the proportion of responses in each category, across all trials.

### Analyses and results

**Overview.** We organized the results section based on the categories defined above, with the aim of discerning the effect of correction strategies on participants’ reliance on discredited information. We begin with an examination of instances where participants endorsed only the misinformation option (i.e., no update), as that indicates a continued reliance on discredited information. By contrasting these patterns between participants in the experimental and no correction baseline conditions, we ascertained the effectiveness of the correction strategies. Specifically, lower misinformation endorsement by experimental participants would indicate effective correction. Next, we investigated whether the CIE was eliminated under any of our correction conditions. If experimental participants performed similarly to the no (mis)information baseline participants, it would suggest that experimental participants were behaving as though they have never encountered the misinformation. Finally, we took advantage of our experimental procedure and took a more nuanced look at the updating processes: discounting discredited information and integrating alternative information.

**Fig. 7:**
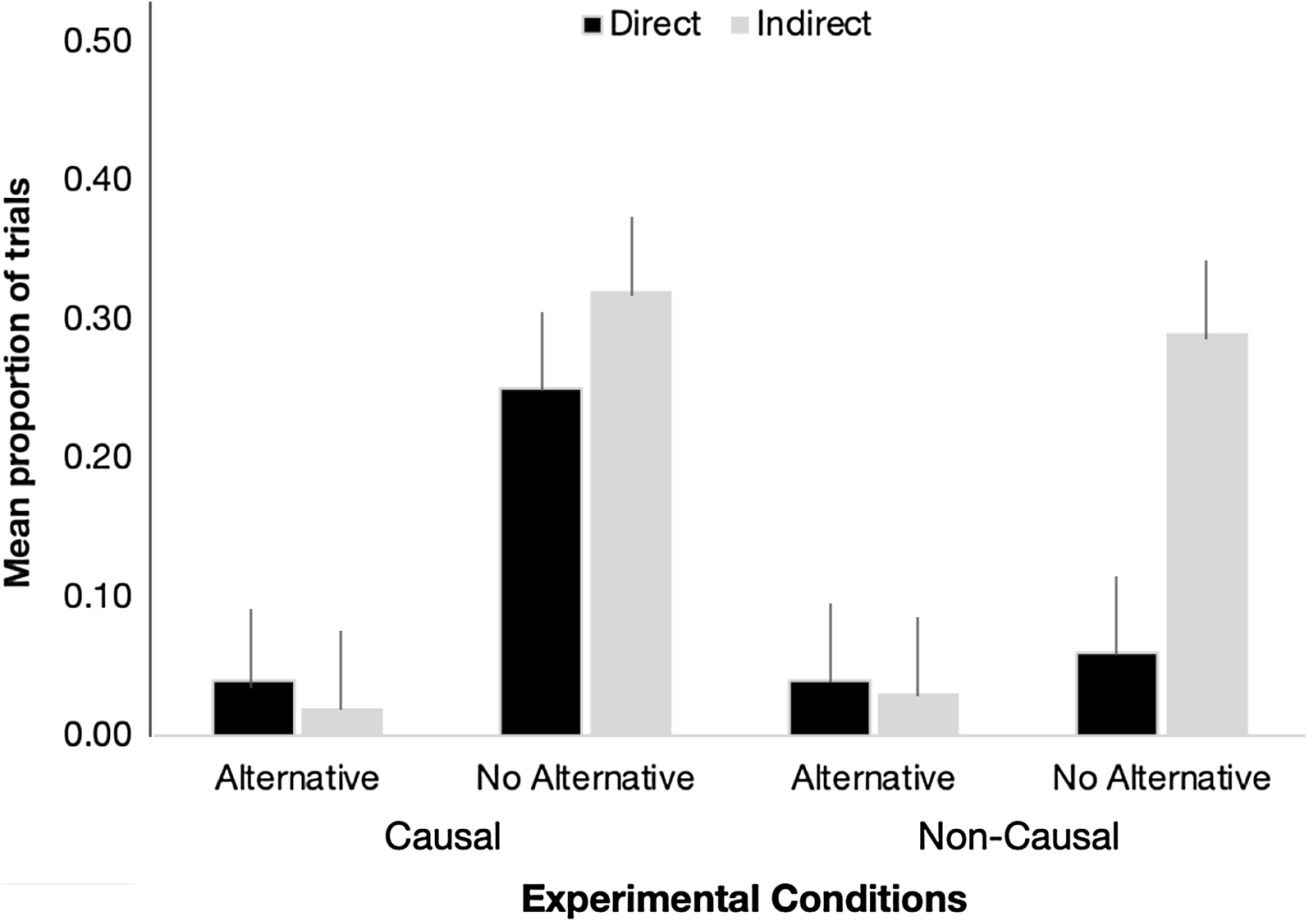
Mean proportion of trials on which experimental participants endorsed only the misinformation option in Experiment 2. Error bars indicate standard error of the mean

**Continued influence effect (i.e., no update).** Figure 7 presents the mean proportion of trials when experimental participants selected only the misinformation option, with a higher proportion reflecting a stronger CIE. To examine whether the effects of alternative provision and correction directness differ for causal and non-causal misinformation, we conducted a 2 (alternative) × 2 (directness) × 2 (causality) ANOVA on proportion of CIE responses. We found a significant main effect of Alternative, *F*(1, 140) = 33.426, *p* < .001, *η*^2^_p_ = .19, and a significant main effect of directness, *F*(1, 140) = 4.222, *p* = .042, *η*^2^_p_ = .03. However, both of these main effects were qualified by a significant alternative x directness interaction, *F*(1, 140) = 5.726, *p* = .018, *η*^2^_p_ = .04. All other effects failed to reach significance (all *p*’s > .05).

Probing of the simple effects revealed the following patterns. (a) When an alternative was offered in the correction statement, further targeting of the misinformation did not appear to affect the proportion of CIE responses, where the comparison failed to reach significance with null hypothesis testing (direct alternative *M* = 0.04, SD = .12 vs. indirect alternative *M* = 0.02, SD = 0.08, *t*(71) = 0.466, *p* = .643, *η*^2^ = .00). This pattern was confirmed with equivalence testing, which showed that these conditions were statistically equivalent, *t*(65) =  − 1.65, *p* = .05. (b) When an alternative account was not offered, however, directly referencing the invalidated information in the correction lowered the CIE, relative to when the reference was indirect. This pattern was supported by an independent sample t test between no alternative direct (*M* = 0.15, SD = 0.25) and no alternative indirect (*M* = 0.31, SD = 0.31) conditions, *t*(73) =  − 2.411, *p* = .018, η^2^ = .07. (c) When the misinformation was directly targeted in the correction statement, the provision of an alternative account significantly reduced participants’ propensity to select only the misinformation option, as evidenced by an independent samples t test (direct alternative vs. direct no alternative, *t*(75) =  − 2.565, *p* = .012, *η*^2^ = .08). (d) This pattern was also observed when the misinformation was not directly targeted in the correction statement (indirect alternative vs. indirect no alternative, *t*(69) =  − 5.178, *p* < .001, *η*^2^ = .28. These patterns are identical to those found in Experiment 1A.

**Explicit endorsement of misinformation.** We included two multiple-choice questions that inquired about the cause and spread of the fire and focused on cases where participants selected only the misinformation option. As discussed earlier, selecting only the misinformation option suggested complete failure to update. We calculated the percentage of participants in the causal conditions endorsing only the “clogged dryer vents” option: direct alternative = 10.5% (*n* = 2), direct no alternative = 36.8% (*n* = 7), indirect alternative = 5.9% (*n* = 1), indirect no alternative = 44.4% (*n* = 8). Similarly, we calculated the percentage of participants in the non-causal conditions selecting only the “paint store” option: direct alternative = 5.3% (*n* = 1), direct no alternative = 40.0% (*n* = 8), indirect alternative = 11.1% (*n* = 2), indirect no alternative = 77.8% (*n* = 14). These patterns mirrored those observed in the probe questions (see Fig. 7).

**Testing for correction effectiveness.** Next, we sought to determine the effectiveness of the correction statements by conducting planned contrasts between each experimental condition against the no correction baseline condition. As shown in Table 7, the contrast between indirect no alternative and no correction baseline (*M* = 0.29, SD = 0.27) conditions^3^ failed to reach significance using null hypothesis testing. We verified this pattern with the equivalence test and found that the two conditions were indeed statistically equivalent, *t*(52) =  − 1.82, *p* = .037. Similar to what we found in Experiment 1A, the indirect no alternative correction statement was ineffectual.

**Table 7:** Summary of Contrast Analyses to Test for Correction Effectiveness in Experiment 2. Contrast Values Refer to Difference in Mean Proportion of (Mis)information Responses Between an Experimental Condition and the No Correction Baseline Condition

Contrast	Direct alternative	Direct no alternative	Indirect alternative	Indirect no alternative	No correction baseline	Contrast value	*t(161), p *value, eta^2^
1	− 1	0	0	0	1	0.26	4.305, < .001, .10
2	0	− 1	0	0	1	0.14	2.395, .018, .03
3	0	0	− 1	0	1	0.27	4.438, p < .001, .11
4	0	0	0	− 1	1	− 0.01	− 0.226, .821, .00

One-way ANOVA, *F*(4, 161) = 13.183, *p* < .001

**Testing for potential elimination of continued influence.** The analyses thus far focused on cases where participants continued to be influenced by discredited information. We next asked whether any of our correction conditions resulted in the elimination of the CIE by contrasting each experimental condition with the no (mis)information baseline condition.

Table 8 presents a summary of the contrast analyses of each experimental condition against the no (mis)information baseline condition (*M* = 0.03, SD = 0.07).^4^ Although two contrasts failed to reach significance using null hypothesis testing, equivalence testing revealed a more nuanced pattern. We confirmed equivalence between the direct alternative and no (mis)information baseline conditions, *t*(60) =  − 2.02, *p* = .024. However, the contrast between indirect alternative and no (mis)information baseline conditions was not equivalent, *t*(55) = 1.56, *p* = .064. Taken together, this suggests that CIE elimination was attained only by individuals who encountered a correction statement that directly targeted the misinformation and included an alternative, replicating the pattern that we observed in Experiment 1B.

**Table 8:** Summary of Contrast Analyses to Test for CIE Elimination in Experiment 2. Contrast Values Refer to Difference in Mean Proportion of (Mis)information Responses Between an Experimental Condition and the No (Mis)information Baseline Condition

Contrast	Direct alternative	Direct no alternative	Indirect alternative	Indirect no alternative	No (mis)information baseline	Contrast value	*t(167), p *value, eta^2^
1	1	0	0	0	− 1	0.004	0.069, .945, .00
2	0	1	0	0	− 1	0.12	2.287, .023, .03
3	0	0	1	0	− 1	− 0.008	− 0.151, .880, .00
4	0	0	0	1	− 1	0.27	5.222, < .001, .14

One-way ANOVA, *F*(4, 167) = 12.965, *p* < .001

**Fig. 8:**
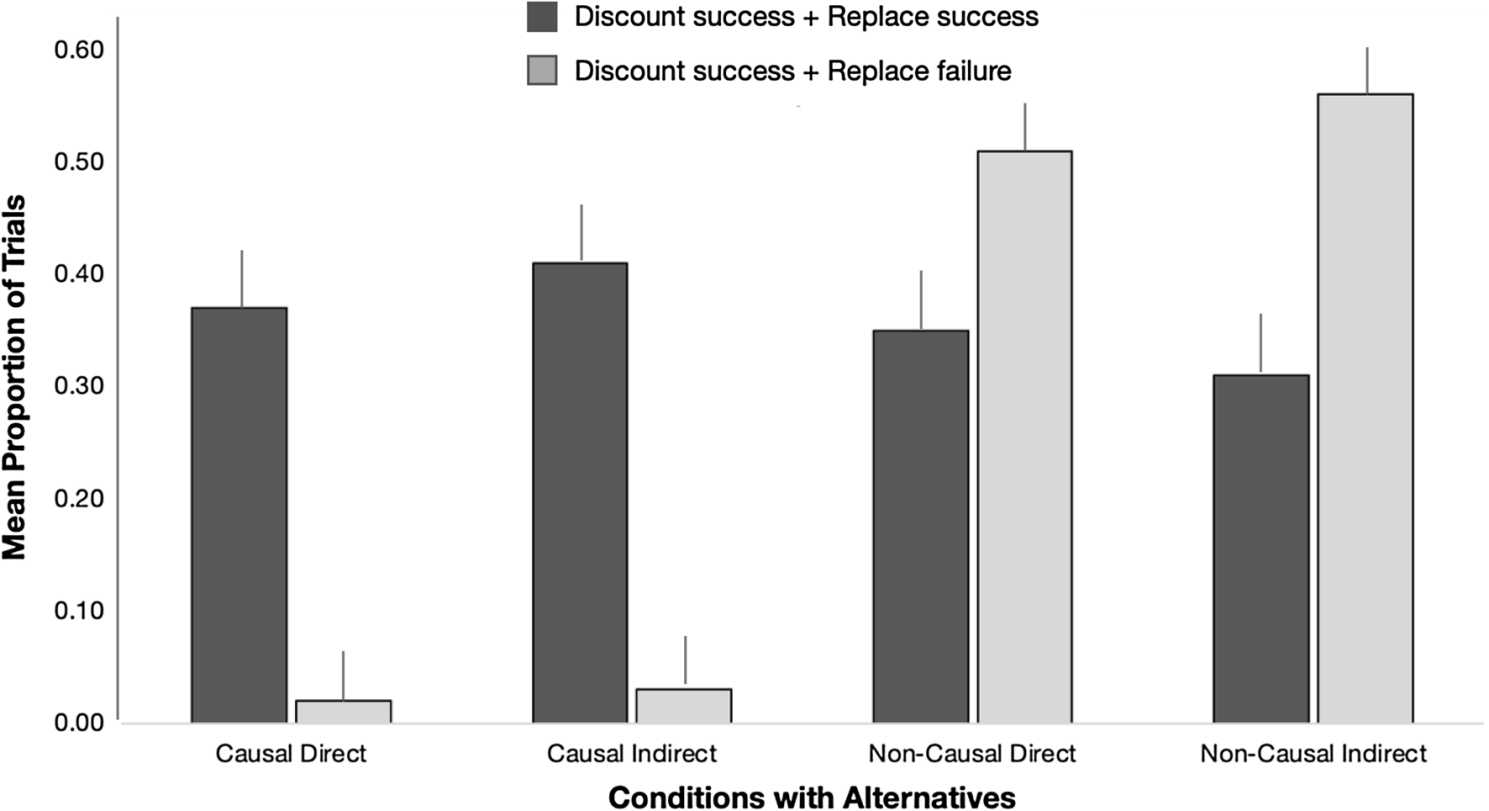
Mean proportion of trials on which participants in different alternative conditions achieved full outdating (darker bars) versus only successful discounting (lighter bars). Error bars indicate standard error of the mean

**Updating processes.** Next, we examined the component processes of mental model updating by considering the relative success of misinformation discounting and alternative information integration. As described earlier, full outdating entails successful discounting of the invalidated information and replacing it with the provided alternative information, whereas partial updating would involve discounting success but replacement failure. To do so, we calculated the proportion of trials when participants endorsed only the alternative option (i.e., full outdating) and the proportion of trials where they selected neither option (i.e., successful discounting but failed replacement). We restricted these analyses to individuals who received an alternative, as full outdating success can only be assessed in those participants. Figure 8 summarizes these results.

We conducted a 2 (outdating success: full vs partial) × 2 (causality) × 2 (directness) mixed ANOVA on mean proportion of trials, with outdating success as a within-subject factor. We found a significant causality main effect, *F*(1, 69) = 89.876, *p* < .001, *η*^2^_p_ = .57, and a significant outdating main effect, *F*(1, 69) = 4.669, *p* = .034, *η*^2^_p_ = .06, but the main effects were qualified by a significant outdating success x causality interaction, *F*(1, 69) = 58.451, *p* < .001, *η*^2^_p_ = .46. All other effects failed to reach significance, all *p*’s > 0.40.

Probing of the simple effects revealed that when the misinformation was causal, participants were more likely to complete both outdating processes compared to achieving discounting success and replacement failure, *t*(35) = 11.550, *p* < .001, η^2^ = .79. In contrast, when the misinformation was non-causal, the opposite pattern was observed, where participants were less likely to achieve full outdating, relative to discounting success and replacement failure, *t*(36) = -− 3.075, *p* = .004, *η*^2^ = .21. Furthermore, although the group difference in full outdating did not reach significance, *t*(71) = 1.218, *p* = .227, *η*^2^ = .02, non-causal participants were significantly more likely to fail at replacing the gap with the alternative information than causal participants, *t*(71) = 13.699, *p* < .001, *η*^2^ = .73.

**Competing representations.** For individuals who received an alternative in the correction statement, another assumed manifestation of partial updating is when both representations are concurrently active and compete for endorsement. To evaluate whether causality and directness impacted such competition, we conducted a 2 (causality) × 2 (directness) ANOVA on the proportion of trials when participants selected both the misinformation and the alternative options. Descriptives for the four conditions are: causal direct alternative *M(*SD*)* = 0.58 (0.19), causal indirect alternative *M(*SD*)* = 0.54 (0.25), non-causal direct alternative *M(*SD*)* = 0.11 (0.13), and non-causal indirect alternative *M(*SD*)* = 0.11 (0.22).

We found that causal subjects experienced significantly greater competition (*M* = 0.56, SD = 0.22) than non-causal subjects (*M* = 0.11, SD = 0.18), a pattern confirmed by a significant main effect of causality, *F*(1, 69) = 91.815, *p* < .001, *η*^2^_p_ = .57. Neither the directness main effect nor the causality × directness interaction reached significance, both *F’s* < 1.

**Successful discounting in the absence of alternative provision.** Here, we focused on discounting success for participants in the no alternative conditions. Since these participants never received the alternative information, replacement success evaluation is not feasible. To determine whether causality and directness impacted discounting likelihood, we conducted a 2 (causality) × 2 (directness) ANOVA on the proportion of trials when individuals did not endorse the misinformation option. Descriptives for the four conditions are: causal direct no alternative *M(*SD*)* = 0.26 (0.23), causal indirect no alternative *M(*SD*)* = 0.21 (0.25), non-causal direct no alternative *M(*SD*)* = 0.88 (0.14), and non-causal indirect no alternative *M(*SD*)* = 0.65 (0.22).

We found that discounting success was affected by causality, such that it was easier to discount the misinformation when it was non-causal (*M* = 0.77, SD = 0.22) relative to when it was causal (*M* = 0.24, SD = 0.24), *F*(1, 71) = 112.662, *p* < .001, *η*^2^_p_ = .61. Furthermore, participants who received a correction statement that referenced the misinformation directly were better able to discount the misinformation (*M* = 0.58, SD = 0.37) than those who received correction statements that indirectly targeted the misinformation (*M* = 0.43, SD = 0.32), *F*(1, 71) = 8.238, *p* = .005, *η*^2^_p_ = 0.10. Finally, the causality × directness interaction failed to reach significance, *F*(1, 71) = 3.462, *p* = .067, *η*^2^_p_ = 0.05.

**Another conceptualization of the CIE.** As described earlier, we operationalized CIE as uncontroverted reference to the misinformation (i.e., referencing only the misinformation in the response). Although this definition is consistent with several classic (e.g., Johnson & Seifert, 1994; Wilkes & Leatherbarrow, 1988) and recent (e.g., O’Rear & Radvansky, 2020) studies on the topic, other researchers have opted for a broader definition. Specifically, some researchers consider any mention to the misinformation (regardless of other accompanying references) as evidence of the persistence of misinformation (e.g., Ecker et al., 2010, 2011a, b). While the former definition includes only the cases where there was no evidence of mental model updating, the latter definition encompasses these egregious cases along with instances of partial updating. In sum, while both definitions represent instances of misinformation persistence, they may indicate varying degrees of influence. In order to connect these conceptualizations of the CIE, we calculated a “total misinformation” proportion score that encompassed all instances of misinformation endorsement (see Table 9).

**Table 9:** Means (standard deviations) of total misinformation proportion for all Experiment 2 conditions

**Total misinformation score** = Misinformation only and misinformation w/alternative endorsements				
	Causal		Non-Causal	
	Alternative	No Alternative	Alternative	No Alternative
Direct	**0.62 (0.20)** = 0.04 + 0.58	**0.74 (0.23)** = 0.25 + 0.49	**0.15 (0.14)** = 0.04 + 0.11	**0.12 (0.14)** = 0.06 + 0.06
Indirect	**0.56 (0.23)** = 0.02 + 0.54	**0.79 (0.25)** = 0.32 + 0.47	**0.14 (0.22)** = 0.03 + 0.11	**0.35 (0.22)** = 0.29 + 0.06

A 2 (alternative) × 2 (causality) × 2 (directness) ANOVA on the total misinformation score revealed a significant main effect of alternative, *F*(1, 140) = 15.574, *p* < .001, *η*^2^_p_ = .10, where participants in the alternative conditions were significantly less likely to endorse misinformation (*M* = 0.36, SD = 0.30) than participants in the no alternative conditions (*M* = 0.49, SD = 0.35). A significant main effect of causality, *F*(1, 140) = 201.767, *p* < .001, *η*^2^_p_ = .59, revealed that respondents in the causal conditions (*M* = 0.68, SD = .24) were significantly more likely to select the misinformation option than subjects in the non-causal conditions (*M* = 0.18, SD = 0.20). Finally, we also observed a significant alternative × directness interaction, *F*(1, 140) = 6.174, *p* = .014, *η*^2^_p_ = .04. Probing of the interaction revealed that the only pairwise comparison that reached significance was indirect alternative (*M* = 0.34, SD = 0.31) versus indirect no alternative (*M* = 0.57, SD = 0.32), *t*(69) = − 3.018, *p* = .004, *η*^2^ = .12.

Finally, we conducted contrast analyses to examine the effectiveness of each correction strategy and whether the CIE was eliminated in any of the conditions. We did so by first calculating a total (mis)information score for the baseline conditions, by including all instances of (mis)information endorsement. We then compared each experimental condition against the no correction baseline condition (causal *M* = 0.87, SD = 0.17; non-causal *M* = 0.37, SD = 0.28) and the no (mis)information baseline condition (causal *M* = 0.62, SD = 0.26; non-causal *M* = 0.12, SD = 0.14) to assess correction effectiveness and CIE elimination, respectively.^5^ Equivalence testing confirmed equivalent broad CIE scores between the no (mis)information baseline and the causal direct alternative conditions and also between the no (mis)information baseline and the non-causal direct no alternative conditions. In addition, equivalence testing failed to reveal any experimental condition that has an equivalent total CIE score as the no correction baseline condition. Thus, CIE reduction was observed in all conditions.

## Experiment 2 Discussion

In Experiment 2, we investigated how the interplay of alternative provision and misinformation targeting in a correction statement was affected by the centrality of the misinformation. We took a relatively novel approach by using close-ended questions (see also Connor Desai & Reimers, 2019; Ecker et al., 2020a, 2020b), which afforded the ability to identify the component processes that may contribute to the continued influence effect. Furthermore, we examined two different conceptualizations of the CIE that are used in the literature: an operationalization that reflects holistic preservation of the misinformation (i.e., when participants select only the misinformation option) and a broader definition that indexes holistic preservation of misinformation and instances of misinformation maintenance alongside an inclination to consider alternative information (i.e., endorsing the misinformation option, either in isolation or along with the alternative). We posit that these conceptualizations (narrow and broad) represent varying degrees of continued influence and propensity to update the mental model. The former indexing complete resistance to update, whereas the latter documenting partial updating and consideration of alternative information.

We consider our findings in the context of the situation model framework, which asserts that as a narrative unfolds, a dynamic mental model that represents the overall meaning of the narrative is developed (Bailey & Zacks, 2015; Bower & Morrow, 1990; Johnson & Seifert, 1994; Johnson-Laird, 2012; Lewandowsky et al., 2012; van Oostendorp & Bonebakker, 1999; Wilkes & Leatherbarrow, 1988). As information is weaved together, a coherent narrative is formed. When elements of the established mental model are called into question, such as by a correction statement, narrative coherence is temporarily disrupted, and comprehension is negatively affected. To understand how misinformation may continue to exert its influence, we first consider three ways in which coherence can be restored and how our data reflect these different possibilities.

First, readers could reject the correction and elect to retain the existing mental model (i.e., no update, holistic retention of misinformation), which would represent the most egregious cases of continued influence. This approach is captured by the narrow CIE measure, where participants endorsed only the misinformation option. We found that both alternative provision and misinformation targeting in a correction statement work in conjunction to influence the CIE. We found that participants are most likely to wholly retain the misinformation when the correction statement neglects to provide an alternative and also fails to specify the misinformation (i.e., indirect no alternative). In fact, such a correction is so ineffectual that those participants endorsed the misinformation option as frequently as baseline participants who never received a correction statement (replicating results from Experiment 1A). In contrast, those who encountered a correction statement that directly targets the misinformation and provides an alternative (i.e., direct alternative condition) rarely insisted on the misinformation and behaved similarly to those individuals who never encountered the misinformation (replicating the finding from Experiment 1B).

Second, readers could engage in partial updating, by maintaining both the original information and corrected content as viable units in their mental models. We suggest that this represents a state of indecision, where the reader hesitates to discount the misinformation but also expresses readiness to consider viable alternatives. When the corrected content is embedded within the correction statement (i.e., alternative conditions), we found greater instances of co-activations of original and alternative information for causal than non-causal misinformation (see “competing representations” sub-section of the Results). Based on the text comprehension literature, we posit that causal misinformation is more central to the narrative than non-causal misinformation, and as such, readers may be reluctant to displace such a central piece of information based on a single correction statement. This reluctance may be exacerbated by the fact that the alternative information and the original causal misinformation are mutually exclusive (i.e., the fire is caused by either clogged dryer vents or faulty electrical panel), which means replacement would result in a substantive change in the overall narrative structure. It is conceivable that until further clarifying information is provided, readers would rather take the intermediate step of keeping both pieces of information active. This overall pattern aligns with the notion that the memory trace for the misinformation may linger and compete with the newly encoded alternative information, and strength of the residual activation is partially determined by the centrality of the misinformation (e.g., Ayers & Reder, 1998; Ecker et al., 2011a, b; Gordon et al., 2019; Kendeou & O’Brien, 2014; Kendeou et al., 2019). Future studies that explore individual differences in decision criterion and other contextual factors that may impact these judgments will be fruitful. Another factor that may be at play here is that both causes of the fire (i.e., clogged dryer vents and faulty electrical panel) are equally plausible. Future work that investigates the role of plausibility in the misinformation and the corrected content will be crucial. Perhaps less plausible misinformation/corrected content (e.g., isolated cyber attack at the laundromat’s electrical grid) will result in less ambivalence in mental model updating.

When we combine the two approaches discussed thus far, we arrive at the basis of the broad CIE measure, which includes all references to misinformation. Under the broad measure, the impact of causality emerged again, where participants in the causal condition had higher CIE scores than those in the non-causal conditions. We believe that the crux of the effect of causality stemmed from the cases of partial updating. Building on our earlier explanation, we reason that the state of indecision—the combined effect of reluctance to reject invalidated information and readiness to consider new information—extends to cases when the correction statement does not include an alternative. When the correction is presented, regardless of alternative provision, the misinformation is tagged as dubious. As described earlier, when an alternative is embedded within the correction statement, that new information may be maintained alongside the tagged misinformation. However, when an alternative is not part of the correction statement, readers may remain in this state of uncertainty without any feasible replacement information for the rest of the narrative. At the time of retrieval, when readers are presented with viable alternatives (by virtue of the close-ended questions), they readily endorse those possibilities as a way to restore coherence post hoc. Thus, although the underlying processes that led to the simultaneous endorsement of misinformation and alternative options differ between the alternative and no alternative conditions, the functional outcome of ambivalence is the same. One avenue of future investigation is to introduce a delayed retrieval phase. It will be of interest to evaluate whether the act of endorsing a realistic alternative that became available post-encoding would result in mental model updating and how that might impact the CIE. Such a line of inquiry would also contribute to the broader post-event misinformation literature (e.g., Loftus, 2005). We will return to a related idea in the General Discussion.

It is worth noting that although we observed similar patterns between the narrow and broad CIE measures, such as the joint impact of alternative provision and directness of misinformation targeting, the effect of causality was apparent only under the broad CIE measure. Taken together, our data suggest that causality only plays a role in the updating processes (i.e., misinformation discounting and replacement) and not the maintenance of misinformation. Future studies that systematically investigate these possibilities and the practical implications of the different CIE conceptualizations will be important.

This broader conceptualization of the CIE also resulted in an unexpected finding. Under the broad measure, we found two conditions that resulted in CIE elimination: causal direct alternative and non-causal direct no alternative (see contrast analyses under broad CIE). While the former is expected and in alignment with the narrow measure, the latter is unanticipated and counter to the situation model. Although we do not have an explanation at this time, we believe this anomalous and puzzling finding warrants further investigation.

Third, a reader could accept the correction and replace the discredited misinformation with the presented alternative, thereby completing the outdating process.^6^ This possibility can be gleaned from instances of successful discounting and replacement, where we found that participants who encountered an alternative in the correction statements, regardless of causality and directness, were similarly successful in achieving full outdating (see Fig. 8). This pattern complements the observation that alternative provision reliably reduces the CIE. In other words, not only does alternative provision lower the instances of misinformation reliance, but it also promotes replacement.

Thus far, our discussion has focused on narrative coherence restoration. We next turn to situations where coherence remains perturbed. Specifically, we focus on cases where readers discounted the misinformation but failed to replace the narrative gap with the provided alternative (see Fig. 8). We found that participants in the non-causal conditions were more likely to fail at replacement than participants in the causal conditions. This pattern is in line with the observation that non-causal participants were less inclined to endorse viable alternatives, if we assume that consideration of viable alternatives is a precursor to replacement success.

Taken together, our utilization of close-ended questions allows for exploration of these various states of mental model updating, ranging from complete resistance to partial updating and full outdating. Consistent with the extant literature and the situation model (and also data from Experiments 1A and 1B), we found that alternative provision plays a key role in the CIE. Furthermore, although causality does not seem to affect holistic preservation of misinformation, it contributes significantly toward multiple facets of mental updating. Although the effect of misinformation targeting was less consistently observed, we found that under the narrow CIE measure, when a correction statement neither targets the misinformation nor provides an alternative, it was largely ineffectual. Although this finding is compatible with the situation model, another possibility remains.

Closer examination of the indirect no alternative correction statements revealed that their syntactic structure might have led to two different interpretations. Consider the statement “The reporter corrected the initial report about the cause of the fire.” One interpretation (which we intended) was that the cause of the fire, which was part of the initial report, needed to be corrected. This correction was indirect because we did not specifically target the misinformation (i.e., clogged dryer vents), and it did not include an alternative. Another interpretation was that the initial report about the fire needs to be corrected, but what part of the report needs to be corrected remains unspecified. Thus, the two interpretations differed in terms of the relative precision with which the readers can identify the content of the correction. To evaluate the likelihood of these interpretations, we conducted a follow-up study, where participants were randomly assigned to view either the causal indirect no alternative correction statement (*n* = 18, *M* age = 22.8, SD age = 2.4) or the non-causal indirect no alternative correction statement (*n* = 18, *M* age = 21.4, SD age = 2.9). Each statement was presented as an excerpt from a Twitter feed (see Fig. 5). Immediately below the statement was the question, “Based on the above excerpt, what information in the initial report needs to be corrected?” Respondents were asked to select either “Cause of fire” (or “Spread of fire” for the non-causal condition) or “Not sufficient information to say.” In both conditions, a high proportion of participants (Causal = 77.8%, Non-Causal = 72.2%) selected the intended interpretations. Participants who interpreted the statements differently than we intended might have to contend with an even larger narrative gap, which might have exaggerated the inadequacy of the indirect no alternative correction statements. Nonetheless, it should be acknowledged that all participants were alerted to the presence of an error in the report and that the correction statements were relatively more specific in the direct conditions than in the indirect conditions. Future studies that systematically vary the size of the narrative gap (and the resulting narrative coherence disruption) will be of interest. This need for further investigation is reinforced by the observation that under the broad CIE measure, this correction strategy in fact successfully reduced the CIE.

In sum, our findings reveal that mental updating is not an all-or-none process and that the different characterizations of the CIE (narrow vs broad) have important implications on how we measure updating success. Future work that systematically contrasts these conceptualizations and explore potential individual differences that may impact distinct updating processes will be particularly beneficial.

## General Discussion

Given the rapid dissemination of information in today’s world, misinformation is inevitable. Unfortunately, real-world examples and experimental evidence suggest that discredited information continues to affect our behaviors and beliefs. In light of the important practical implications of the continued influence of misinformation, it is crucial to understand how one might minimize the potential negative impact of fake news. Across three experiments, we considered three factors associated with the CIE: whether an alternative was offered at the time of correction, whether the misinformation was targeted in the retraction, and whether the content of the misinformation was central to the unfolding narrative. Although prior studies have examined these factors, the relative contribution of each remains unclear. In addition, by employing a dual baseline approach, we were able to assess both correction effectiveness and CIE elimination. Finally, in Experiment 2, we introduced a relatively novel approach to examine the CIE. By utilizing close-ended responses, we are able to assess the component processes that may underlie the persistence of misinformation, including the representational competition between the discredited information and the alternative and the relative success in replacing the invalidated information with the alternative information. We also considered how the CIE may be manifested under two different conceptualizations: a narrow measure that indexes only the complete preservation of the misinformation and a broad measure that also includes instances where both the misinformation and the alternative information are viable.

We interpreted our findings within the situation model framework (e.g., Bailey & Zacks, 2015; Bower & Morrow, 1990; Ecker et al., 2011a, b; Johnson-Laird, 2012; Lewandowsky et al., 2012; van Oostendorp & Bonebakker, 1999). Across the three experiments (and both narrow and broad measures of the CIE), the most consistent finding is that alternative provision within the correction statement reduced the persistence of misinformation. Although less reliable, we observed some instances of CIE elimination and correction statements that were wholly ineffectual. As reviewed above, CIE elimination is infrequently observed (for noted exception, see Johnson & Seifert, 1994). The scarcity of this finding in the literature may be due to the fact that the CIE is extremely robust and also the possibility that conditions that may produce elimination may depend on the precise conceptualization of the CIE. These differences are worth further scrutiny in future studies. Finally, although causality did not appear to impact the holistic preservation of the misinformation, its key role in the CIE is reflected in a state of ambivalence (i.e., reluctance to reject the misinformation combined with a readiness to consider alternatives) and also in the different processes that support mental model updating.

Although there is some evidence to support the idea that misinformation repetition may result in greater perceived truth (e.g. Dechêne et al., 2010; Fazio et al., 2019; Hasher et al., 1977; see also the backfire effect, e.g., Seifert, 2002, Lewandowsky et al., 2012), the findings on misinformation repetition specifically have been mixed (e.g., Ecker et al., 2017, 2011a, b; Wilkes & Leatherbarrow, 1988). Our results suggest a beneficial effect associated with direct targeting (thereby repeating) of misinformation in the correction statement. Future studies should further consider this issue, as it is a fairly common practice in online journalism (e.g., Time Magazine, 2018; Winter & Ainsley, 2018).

As described earlier, the continued influence of misinformation is typically considered in the context of failure in mental model updating or competing activations of discredited misinformation and alternative information. By utilizing close-ended questions and encouraging participants to select all response options that apply, we evaluated both notions and provided support for both types of models (see Gordon et al., 2019 for a recent attempt to distinguish these models using fMRI). Consistent with the CIE literature, we propose that successful revision of a mental model requires multiple steps, including successful discounting of the obsolete information and replacing the invalidated information with an alternative (Kendeou et al., 2013; O’Brien et al., 2010). When these processes are incomplete, they may manifest themselves as simultaneous maintenance of tagged misinformation and plausible alternative. Thus, our approach complements the extant literature and represents the first step toward further characterizing the possible components that comprise the construct of CIE.

In contrast to open-ended responses, selections in closed-ended questions may rely heavily on familiarity-driven processes, a common issue with recognition memory tasks (e.g., Yonelinas, 2001). However, since both open-ended and close-ended assessments of the CIE yielded largely similar patterns (see also Connor Desai & Reimers, 2019), this may not present a major challenge to our conclusions. Nevertheless, additional studies that directly compare the two types of response modality, and by extension the associated memory retrieval processes of recollection and familiarity, will be instructive.

In addition, the reasoning behind participants’ decision to not endorse a particular response option remains open to interpretation. In our analyses, we interpreted the non-selection of the misinformation option as successful discounting and the non-selection of the alternative option as failure to use the alternative to fill the narrative gap. Another possibility, however, is that subjects did not select those options because of a memory failure. However, given the strong performance on the comprehension questions (causal *M(*SD*)* = 0.93 vs. non-causal *M(*SD*)* = 0.94), this interpretation seems less likely. Furthermore, this issue is not unique to close-ended questions, as it would be a challenge to interpret any omissions in the responses. In future work, it may be instructive to adopt a method that requires participants to make an active choice for each option. That is, instead of asking participants to select all options that apply (e.g., Question 1 in Experiment 2: “Which of the following factor(s) contributed to the fire? (Select all that apply)”), requiring participants to make a yes/no judgment about each response option may address this issue (e.g., Did clogged dryer vents contribute to the fire? Did faulty electrical wiring contribute to the fire?). This approach would be similar to that employed by Connor Desai and Reimers (2019).

Our studies contribute to the CIE literature in several important ways, as noted above. However, several limitations are worth noting. Although our inclusion of three narratives improved generalizability over studies that used only one narrative, it would be beneficial for future studies to include other narrative material as well. Relatedly, most CIE studies that rely on narratives use content that is potentially arousing (e.g., warehouse fire, car accident, theft, burglary), it would be important to extend the current findings to neutral material.

In addition, similar to most studies, we presented and corrected the misinformation in the same testing session as the CIE assessment. In a recent study, Rich and Zaragoza (2020) suggested that the influence of misinformation can change over time, with the CIE having a rebound effect at later time points. As such, it will be of interest to vary the time delay between misinformation/correction and CIE assessment in future work (see Ecker et al., 2020a, 2020b). This is of particular relevance to our understanding of the stability of mitigation strategies. Future studies should focus on whether the combination of strategies we offered will be effective in cases where the spreading of misinformation is particularly rapid and pervasive and in cases where the medium is not a narrative (e.g., infographic).

In sum, although we have offered some answers with our data, many critical questions remain. For example, how might we combat the persistence of misinformation in real-world scenarios, where we are constantly bombarded with misinformation? Is there a point of no return, such as the WMD and vaccine examples described in the literature (Larson et al., 2011; Lewandowsky et al., 2009), where once a critical threshold is reached, no amount of negation can counter the effect? Until we can identify a strategy to stop the spread of fake news, the next best thing is to find ways to mitigate their consequences.

## Data Availability

The datasets used and/or analyzed during the current study are available from the corresponding author on reasonable request.

## Appendix A: Stimuli

Information presented within [brackets] is not presented to participants.

### Experiment 1A: Minibus Accident Story

*Instructions:* You will read a series of tweets about a minibus accident that occurred in Clawson, Michigan, a Detroit suburb. The story was posted via the *Clawson Times Courier’s* Twitter account, and each tweet will be presented on its own page. Please read the story carefully, as we will ask you some questions about it later.

1. On Sunday night, Police received a report from a passing motorist about a serious minibus accident.
2. The minibus had crashed into a steep embankment near Spring Street and had rolled on its side.

Some of the passengers on board were injured.

[3] A rescue crew was dispatched to the scene immediately upon report of the accident. They arrived at the scene within 10 min.
[4 - **All experimental and No Correction Baseline conditions]**
  Police stated that the passengers on the minibus were a group of elderly people on their way back to their nursing home after a bingo game. The weather was reportedly fine and visibility was good. No other vehicles seem to have been involved.
[4 - **No (Mis)Information Baseline condition]**
  Police stated that the passengers on the minibus were a group of middle school children returning home from a field trip. The weather was reportedly fine and visibility was good. No other vehicles seem to have been involved.
[5] When the rescue crew began evacuation of the minibus, they tried to reach the injured passengers first but found it difficult to tell them apart from the uninjured.
[6] The rescue crew also reported difficulty in evacuating the minibus, even though the exits were clear.
[7] Authorities are desperately trying to reach the passengers’ family members to inform them of the accident. Meanwhile, they have managed to trace the minibus license plate to a rental company.
[8] The crew reported that the rescue efforts were slow and would take several more hours. Bystanders and motorists are advised to avoid the Spring Street area.
[9] Live TV footage from the scene showed uninjured passengers having problems getting up the steep embankment.
[10] Rescue crew can be heard remarking that the uninjured passengers were unable to help in the rescue efforts.
[11 - **Both Baseline conditions]**
  A second statement from the Police confirmed that all passengers were successfully rescued.
[11 - **Direct Alternative]**
  A second statement from the Police corrected the initially reported information about the passengers; the passengers were not elderly people but were in fact middle school children returning home from a field trip.
[11 - **Direct No Alternative]**
  A second statement from the Police corrected the initially reported information about the passengers; the passengers were not elderly people.
[11 - **Indirect Alternative]**
  A second statement from the Police corrected the initially reported information about the passengers; the passengers were in fact middle school children returning home from a field trip.
[11 - **Indirect No Alternative]**
  A second statement from the Police corrected the initially reported information about the passengers.

[12] Passengers with injuries were taken to the nearby St Joseph’s Hospital for treatment.
[13] At the hospital, 3 passengers with more serious injuries had to remain for observation, while the others were discharged after treatment.
[14] Some of the uninjured passengers interviewed at the accident scene remarked on how helpless and scared they felt, and they were grateful for the rescue crew.

### Experiment 1B: Home Burglary Story

*Instructions:* You will read a series of tweets about a home burglary that occurred in Clawson, Michigan, a Detroit suburb. The story was posted via the *Clawson Times Courier’s* Twitter account, and each tweet will be presented on its own page. Please read the story carefully, as we will ask you some questions about it later.

1. On Sunday evening, Police responded to a call made from a home on Acorn St., in a middle-class residential neighborhood.
2. The caller, Mrs. Gallagher, reported hearing the sounds of breaking glass and a car speeding away. She suspected a burglary had taken place at her neighbor’s house.
3. Police arrived within half an hour and began an investigation. The Police tried to contact the homeowner Mr. Emmert, but he was away on vacation. Mrs. Gallagher suggested that the Police contact Mr. Emmert’s son, Brian.
4. After surveying the house, the Police noticed signs of forced entry and a broken window.
5. **All experimental and No Correction Baseline conditions]**
  When Brian arrived, he saw that the house was ransacked, and the garage was empty. Brian stated that in addition to the blue minivan, many other valuable items were missing, including jewelry, antique watches, and cash.
6. **No (Mis)Information Baseline condition]**
  When Brian arrived, he saw that the house was ransacked. Brian stated that many valuable items were missing, including jewelry, antique watches, and cash.
7. The Police Detective informed Brian that the neighborhood has been hit with a number of burglaries recently and noted that they all took place while the homeowners were away.
8. There are no arrests or leads in these cases so far. The Police issued a statement warning the neighbors to be more vigilant and to report suspicious activities.
9. Several neighbors went to Mr. Emmert’s house because they saw flashing Police lights.
10. The group decided to organize a neighborhood watch, given the string of break-ins in their community.
11. After the Police left, the neighbors returned to their homes. Brian called his father to tell him about the burglary and the stolen items.
12. Mr. Emmert was upset and planned to return home early from his trip. Until then, he asked Brian to have the broken window repaired as soon as possible.
13. **Both baseline conditions]**
  After speaking with his father, Brian called the Police to let them know that his father would be calling the Police station later that day.
14. **Direct Alternative]**
  After speaking with his father, Brian had to call the Police immediately to correct the report; the minivan was not stolen. In fact, Mr. Emmert had taken his minivan to the mechanic for repair right before his trip.
15. **Direct No Alternative]**
  After speaking with his father, Brian had to call the Police immediately to correct the report; the minivan was not stolen.
16. **Indirect Alternative]**
  After speaking with his father, Brian had to call the Police immediately to correct the report. In fact, Mr. Emmert had taken his minivan to the mechanic for repair right before his trip.
17. **Indirect No Alternative]**
  After speaking with his father, Brian had to call the Police immediately to correct the report.

[13] When he got home, Mr. Emmert contacted his insurance company about the loss and hired a security company to install a surveillance system.
[14] After weeks of investigation, the Police arrested excon Dan Fowler and his accomplice, who had tried to sell some of the the stolen goods. The detectives recovered the stolen items and are now looking for similarities between this case and the other recent break-ins in the neighborhood.

### Experiment 2: Fire Story

*Instructions:* You will read a series of tweets about a fire that occurred in Clawson, Michigan, a Detroit suburb. The story was posted via the *Clawson Times Courier’s* Twitter account, and each tweet will be presented on its own page. Please read the story carefully, as we will ask you some questions about it later.

1. On Monday afternoon, Police received a call from a homeowner on Acorn Lane, which is located in a residential neighborhood near downtown Detroit.
2. The caller, Mrs. Gallagher, reported that the strong wind gusts were blowing thick smoke, along with the smell of burning material, toward her neighborhood.
3. After speaking with the Police, Mrs. Gallagher joined a group of neighbors who had congregated on the sidewalk. She shared with the neighbors that the Police had just dispatched emergency vehicles to the scene. Within a few minutes, the group heard sirens in the distance and counted three fire engines.
4. The local TV station confirmed that the scene of the fire was the recently renovated and expanded laundromat on Front Street. The reporter said there was strong heat coming from the fire, and the firefighters had to wear extra protective gear.
5. **All experimental and No Correction Baseline conditions]**
  Several eyewitnesses reported seeing smoke from one of the dryers, adding that the dryer vents could have been clogged because the laundromat attendant often neglects to clear them. They also stated that the fire had begun to spread to the neighboring paint store.
6. **No (Mis)Information Baseline condition]**
  Several eyewitnesses reported seeing sparks at the electrical panel, adding that the recent renovations included rewiring for the new machines. They also stated that the fire had begun to spread to the neighboring auto mechanic shop.
7. Another neighbor chimed in and said that he just spoke with the laundromat owner the day before, and the owner said he was pleased with the new business generated by the expansion.
8. Some of the neighbors expressed how much they liked the new changes. The washers seemed more efficient, and the waiting area is much more comfortable.
9. After discussing other changes in the neighborhood, the neighbors returned to their respective homes to watch the live TV coverage.
10. Live TV footage showed the reporter interviewing the Police Captain, who said that two employees suffered injuries and were being treated at the local hospital.
11. The reporter added that the firefighters appeared to have the flames under control.
12. **Both baseline conditions]**
  The camera then showed a crowd of bystanders, watching the blaze. Several bystanders were taking photos with their cell phones.
13. **Causal, Direct Alternative]**
  The camera then showed a crowd of bystanders, watching the blaze. The reporter corrected the initial report about the cause of the fire. It was not caused by clogged dryer vents. The fire was actually caused by faulty electrical wiring in the laundromat.
14. **Causal, Direct No Alternative]**
  The camera then showed a crowd of bystanders, watching the blaze. The reporter corrected the initial report about the cause of the fire. It was not caused by clogged dryer vents.
15. **Causal, Indirect Alternative]**
  The camera then showed a crowd of bystanders, watching the blaze. The reporter corrected the initial report about the cause of the fire. The fire was actually caused by faulty electrical wiring in the laundromat.
16. **Causal, Indirect No Alternative]**
  The camera then showed a crowd of bystanders, watching the blaze. The reporter corrected the initial report about the cause of the fire.
17. **Non-causal, Direct Alternative]**
  The camera then showed a crowd of bystanders, watching the blaze. The reporter corrected the initial report about the spread of the fire. While the fire did spread to another business, it turned out it was not the neighboring paint store. The fire had instead spread to the auto mechanic repair shop next door.
18. **Non-causal, Direct No Alternative]**
  The camera then showed a crowd of bystanders, watching the blaze. The reporter corrected the initial report about the spread of the fire. While the fire did spread to another business, it turned out it was not the neighboring paint store.
19. **Non-causal, Indirect Alternative]**
  The camera then showed a crowd of bystanders, watching the blaze. The reporter corrected the initial report about the spread of the fire. The fire had instead spread to the auto mechanic repair shop next door.
20. **Non-causal, Indirect No Alternative]**
  The camera then showed a crowd of bystanders, watching the blaze. The reporter corrected the initial report about the spread of the fire.

[12] While speaking with the reporter, the Police Captain received news from the hospital that the injured employees have been discharged.
[13] The Police Captain added that a team will begin an investigation immediately and will interview all involved parties.
[14] A few weeks after the fire, the laundromat owner took out an ad in the local newspaper to announce a grand re-opening the following month.

## Appendix B: Probe questions to assess CIE and comprehension

### Experiment 1A: Minibus story

Questions that assess CIE

1. Why do you think it was difficult getting both the injured and uninjured passengers out of the minibus?
2. Which family members of the passengers’ are authorities most likely to contact to inform them about the accident?
3. Why do you think it was difficult getting the uninjured passengers up the embankment?
4. Why do you think the uninjured passengers were unable to help with the rescue efforts?
5. Why do you think some passengers were injured while others were not?
6. Why did the uninjured passengers feel helpless and dependent on the rescue crew?

*Forced choice questions that assess overall comprehension (correct answer in bold).*

1. When did the accident occur?
  - Monday.
  - Wednesday.
  - **Sunday**
2. Who reported the accident to the Police?
  - **A motorist driving past**
  - The bus driver.
  - One of the passengers.
3. What was the weather like on that day?
  - Heavy snow.
  - Foggy.
  - **Clear and good visibility**
4. How many injured passengers were kept for observations?
  - 0
  - **3**
  - 10.

*Forced choice question that explicitly assessed retention of misinformation (answer that represents misinformation in bold).*

1. How old were the passengers?
  - Young.
  - Middle-aged.
  - **Elderly**

**Experiment 1B: Home burglary story**

*Questions that assess CIE*

1. Where would the burglars take the stolen items to be sold?
2. What was the getaway vehicle driven by the burglars?
3. Was the garage door open when the Police arrived at the scene?
4. How could the neighbors be more vigilant to prevent burglaries like that of the Emmerts?
5. What stolen items would Mr. Emmert need to replace upon his return?
6. Where would Mr. Emmert go to pick up his minivan when he returned?

*Forced choice questions that assess overall comprehension (correct answer in bold).*

1. When did the burglary occur?
  - Monday.
  - Wednesday.
  - **Sunday**
2. Where was Mr. Emmert when the burglary took place?
  - **On vacation**
  - At work.
  - In the hospital.
3. Were the burglars caught?
  - **Yes**
  - No.
4. What action did the neighbors decide to take?
  - Host a fundraiser to help Mr. Emmert pay for the damages.
  - **Organize a neighborhood watch**
  - Talk to the Police Chief about increasing Police patrol in the neighborhood.

*Forced choice question that explicitly assessed retention of misinformation (answer that represents misinformation in bold).*

1. Did the burglars steal Mr. Emmert’s minivan?
  - Yes.
  - **No**

### Experiment 2: Fire Story

*Questions that assess CIE*

1. Which of the following factor(s) contributed to the fire? (Select all that apply)
  - Cans of paint and paint thinner
  - Tires and gas cans
  - Clogged dryer vents
  - Faulty electrical wiring.
  - Strong wind gusts.
  - None of the above.
2. Which of the following individual(s) should the fire investigator interview and inform? (Select all that apply)
  - Owner of the nearby paint store
  - Owner of the nearby auto mechanic repair shop
  - Laundromat attendant
  - Electrician who worked on the laundromat renovation
  - Eyewitnesses
  - None of the above
3. Which of the following location(s) should the fire investigator focus on during evidence collection? (Select all that apply)
  - The nearby paint store
  - The nearby auto mechanic repair shop
  - The section of the laundromat where the dryers are located
  - Electrical panel in the laundromat
  - Mrs. Gallagher’s house
  - None of the above
4. Which of the following piece(s) of information should the fire investigator include in the final report? (Select all that apply)
  - The large shipment of paint recently delivered to the nearby paint store.
  - The technician at the nearby auto mechanic shop often leaves the machines on overnight
  - The safety inspection report of the laundromat’s dryer vents
  - Photographs of the laundromat’s electrical wiring
  - New furniture in the laundromat
  - None of the above
5. Which of the following event(s) would likely to occur as a result of the fire? (Select all that apply)
  - The dismissal of the laundromat attendant
  - Owner of the nearby auto mechanic shop will need to file a claim with his insurance company
  - Owner of the nearby paint store will need to take out a small business loan
  - A qualified electrician will be hired to inspect the rest of the wiring at the laundromat
  - Drivers will experience traffic delay along Front Street
  - None of the above
6. Which of the following individual(s) would likely be held financially and/or legally responsible for the property damage caused by the fire? (Select all that apply)
  - Owner of the nearby paint store.
  - Owner of the nearby auto mechanic repair shop.
  - Laundromat attendant.
  - Electrician who worked on the laundromat renovation.
  - Mrs. Gallagher.
  - None of the above.

Forced choice questions that assess overall comprehension (correct answer in bold).

1. When will the laundromat reopen?
  - **The following month**
  - In 6 months.
  - No plan to reopen.
2. How many fire engines were dispatched to the location?
  - 1
  - **3**
  - 5
3. Who reported the fire to the Police?
  - Mrs. Riley.
  - **Mrs. Gallagher**
  - Mrs. Williams.
4. Where were the injured employees treated?
  - At the scene.
  - In the ambulance.
  - **At the local hospital**
5. Where was the fire?
  - **Laundromat**
  - Restaurant.
  - Clothing store.

Forced choice question that explicitly assessed retention of misinformation (answer that represents misinformation in bold).

1. To what location(s) did the fire spread? (Select all that apply)
  - Paint store.
  - Auto mechanic repair shop.
  - Mrs. Gallagher’s house.
2. What might have been the cause(s) of the fire? (Select all that apply)
  - Clogged dryer vent.
  - Faulty electrical wiring.
  - Arson.

## Abbreviations

ANOVA: Analysis of variance
CIE: Continued influence effect
M: Mean
SD: Standard deviation

## Footnotes

1 While the rarity of the complete elimination of the CIE is a testament to the robustness of the effect, it should also be noted that not all studies included the comparison condition (i.e., a baseline condition where subjects never experienced the misinformation) that is necessary to test for elimination. Nonetheless, even when the no misinformation baseline comparison condition is present, the CIE is rarely eliminated (for a review, see Chan et al., 2017).

2 One might question whether the difference in the proportion of individuals who endorsed the (mis)information between the two experiments might have stemmed from the fact that the minibus question had three response choices, whereas the burglary question had only two options, thereby increasing the chances of stolen minivan endorsement. This potential issue is addressed in Experiment 2.

3 Since the ANOVA on CIE for the probe questions did not reveal a causality main effect (nor did it interact with other factors), we collapsed across causality when reporting the baseline mean. For the sake of complete reporting, we report the means for the baseline conditions by causality here: Causal *M(SD)* = 0.29 (0.31), Non-Causal *M*(SD) = 0.30 (0.23).

4 Since the ANOVA on CIE for the probe questions did not reveal a causality main effect (nor did it interact with other factors), we collapsed across causality when reporting the baseline mean. For the sake of complete reporting, we report the means for the baseline conditions by causality here: Causal *M(SD)* = 0.03 (0.07), Non-Causal *M(SD)* = 0.03 (0.08).

5 It is important to note that the contrast analyses based on the broad measure were configured differently than those on the narrow measure. In the narrow measure analyses, the scores were collapsed across causality because the factor did not reach significance in the omnibus test. However, since causality exerted significant influence in the broad measure analyses, causal and non-causal conditions remained distinct in the contrast analyses. Thus, caution must be exercised when directly comparing the contrast analyses across the two measures.

6 It should be noted that this possibility is distinct from the CIE elimination contrast analyses reported earlier (though they are complementary). The contrast analyses are based on continued endorsement of the misinformation, whereas the outdating process focuses on the discounting (i.e., *not* endorsing) of the misinformation and selection of the replacement information.
